# Using marginal standardisation to estimate relative risk without dichotomising continuous outcomes

**DOI:** 10.1186/s12874-019-0778-9

**Published:** 2019-07-29

**Authors:** Ying Chen, Yilin Ning, Shih Ling Kao, Nathalie C. Støer, Falk Müller-Riemenschneider, Kavita Venkataraman, Eric Yin Hao Khoo, E-Shyong Tai, Chuen Seng Tan

**Affiliations:** 1 Saw Swee Hock School of Public Health, National University of Singapore, Singapore, Singapore; 2 NUS Graduate School for Integrative Sciences and Engineering, National University of Singapore, Singapore, Singapore; 3 Department of Surgery, Yong Loo Lin School of Medicine, National University of Singapore and National University Hospital System, Singapore, Singapore; 4 Department of Medicine, National University Hospital, Singapore, Singapore; 5 Department of Medicine, Yong Loo Lin School of Medicine, National University of Singapore, Singapore, Singapore; 6 Norwegian National Advisory Unit on Women’s Health, Oslo University Hospital, Oslo, Norway

**Keywords:** Relative risk, Linear models, Logistic models, Dichotomisation, Odds ratio, Hyperglycaemia

## Abstract

**Background:**

Although criticisms regarding the dichotomisation of continuous variables are well known, applying logit model to dichotomised outcomes is the convention because the odds ratios are easily obtained and they approximate the relative risks (RRs) for rare events.

**Methods:**

To avoid dichotomisation when estimating RR, the marginal standardisation method that transforms estimates from logit or probit model to RR estimate is extended to include estimates from linear model in the transformation. We conducted a simulation study to compare the statistical properties of the estimates from: (i) marginal standardisation method between models for continuous (i.e., linear model) and dichotomised outcomes (i.e., logit or probit model), and (ii) marginal standardisation method and distributional approach (i.e., marginal mean method) applied to linear model. We also compared the diagnostic test for probit, logit and linear models. For the real dataset analysis, we applied these analytical approaches to assess the management of inpatient hyperglycaemia in a pilot intervention study.

**Results:**

Although the RR estimates from the marginal standardisation method were generally unbiased for all models in the simulation study, the marginal standardisation method for linear model provided estimates with higher precision and power than logit or probit model, especially when the baseline risks were at the extremes. When comparing approaches that avoid dichotomisation, RR estimates from these approaches had comparable performance. Assessing the assumption of error distribution was less powerful for logit or probit model via link test when compared with diagnostic test for linear model. After accounting for multiple thresholds representing varying levels of severity in hyperglycaemia, marginal standardisation method for linear model provided stronger evidence of reduced hyperglycaemia risk after intervention in the real dataset analysis although the RR estimates were similar across various approaches.

**Conclusions:**

When compared with approaches that do not avoid dichotomisation, the RR estimated from linear model is more precise and powerful, and the diagnostic test from linear model is more powerful in detecting mis-specified error distributional assumption than the diagnostic test from logit or probit model. Our work describes and assesses the methods available to analyse data involving studies of continuous outcomes with binary representations.

**Electronic supplementary material:**

The online version of this article (10.1186/s12874-019-0778-9) contains supplementary material, which is available to authorized users.

## Background

Dichotomisation of continuous outcomes is common in epidemiology. For example, certain conditions of interest are defined by a continuous variable over or below some threshold, such as, hyperglycaemia is determined by either pre-meal blood glucose (BG) exceeding 7.78 mmol/L or random BG exceeding 10 mmol/L [1, 2]. The nature of the outcome determines the statistical approach taken to analyse the data. For example, linear model and logit model (or logistic regression model) are commonly performed on continuous and binary outcomes respectively. Hence, for a continuous outcome where its binary representation is also widely used, studies have reported findings from both linear and logit models for outcomes with dual representations [3–5].

However, estimates from dichotomised outcomes have large variances [6, 7] and low power [6–10]. Despite of these disadvantages, there are practical reasons for justifying dichotomisation, such as: (a) following practices used in previous research, (b) simplifying analyses or presentation of results, (c) addressing skewed variable, and (d) using clinically significant thresholds [9, 10]. To address the problems associated with dichotomising continuous outcomes, approaches that use the analytical results from continuous outcomes to infer the association between an exposure and the dichotomised outcome have been proposed.

The proposed approaches transform the estimates obtained from continuous outcomes into familiar measures of association for binary outcomes, such as, risk differences, odds ratios (ORs), and relative risks (RRs). The estimates are obtained from applying least squares [11], method of moments [12–14], maximum likelihood [15, 16], or Bayesian [17, 18] estimation method to continuous outcomes. The simplest transformation multiplies a scaling factor to the estimates from the linear model to obtain log-OR [11] but it assumes the errors have a logistic distribution. The Bayesian method allows the distribution of the error to be unspecified [18]. An alternative approach uses the dichotomised marginal means from the linear model for continuous outcomes [12, 13, 15] to obtain the measures of association for binary outcomes and the skew normal distribution [14] has been considered to address potential skewness in the continuous outcomes.

The marginal mean approach estimates probabilities of different exposure levels by assuming an individual having a confounding profiling that corresponds to the mean values of the confounders [19]. When one of the confounders is binary, an individual having a binary confounder that equals to its mean value does not exist in the real world setting. However, when the outcome is continuous, the marginal mean is equivalent to the overall mean of the population in the linear regression model because the model has an identity link. Given that the computation of probabilities for binary outcomes involve non-linear link function (e.g., logit link), marginal standardisation is commonly used to generate probabilities and RRs from the logit model for making inference on the overall study population [19]. Interestingly, when the study of continuous outcomes does not require adjustment for confounders with regression model, for example, in randomised controlled trials, the marginal mean method for estimating one-sample risk and RR from two-sample [12, 15] could be equivalent to the marginal standardisation method under certain assumptions (see Additional file 1 Section 1 for the details).

In this paper, we leverage on the marginal standardisation method for binary outcomes to estimate the RR of dichotomised outcomes using the linear regression model with adjustment for confounders. Extending the marginal standardisation method from binary to dichotomised outcomes becomes apparent when we realise that the logit (or probit) model assumes an underlying latent variable that corresponds to a linear model with standard logistic (or normal) error [20–22], and when the latent variable exceeds some threshold (i.e., dichotomised latent variable) it can be modelled using the logit (or probit) model. As both the logit and probit models are commonly used to model binary outcomes, we extend the marginal standardisation method to linear model to make inference on dichotomised outcomes in two scenarios: logistic and normal error distributions. We compare the marginal standardisation method between regression models for continuous (i.e., linear model) and dichotomised (i.e., logit or probit model) outcomes by comparing the estimates generated from these regression models. Among approaches that avoid dichotomisation, we compare RR estimates that used the marginal standardisation approach for linear model with those from the distributional approach proposed by Sauzet et al. [16] that estimates RR from the marginal mean obtained from the same linear model. We assess the statistical properties of the estimates and diagnostic tests using simulated data. We also apply the various approaches to evaluate the effect of an intervention that aims to improve inpatient management of hyperglycaemia in a pilot study where multiple thresholds were used to represent varying levels of severity in hyperglycaemia.

## Methods

### Statistical models

The commonly used linear model for a continuous outcome has the following structure:

1 $$ {Y}_i={\alpha}_0+{\alpha}_1{E}_i+\sum \limits_{k=1}^p{\alpha}_{k+1}{Z}_{ki}+{\varepsilon}_i $$

where *Y*_*i*_, *E*_*i*_ and *Z*_*ki*_, *k* = 1, …, *p*, are the observed outcome, exposure and confounders for the i-th individual respectively; α_0_ is the intercept and α_1_, ⋯, α_p + 1_ are the slopes; and the error terms, ε_i_s, are assumed to be independent and identically distributed from a normal distribution with mean being 0 and standard deviation (SD) being λ. We call this the normal linear model from henceforth.

Suppose the dichotomised outcome is defined by the continuous outcome exceeding a known threshold, i.e., $\overset{\sim }{Y_i^1}=\mathrm{I}\left({Y}_i>\tau \right)$, where τ denotes the threshold and I(∙) is the indicator function, the dichotomised outcome would correspond to a probit model where the linear predictor of the probit model (LP) is:

2 $$ {LP}_i={\beta}_0+{\beta}_1{E}_i+\sum \limits_{k=1}^p{\beta}_{k+1}{Z}_{ki} $$

where the link function is the inverse cumulative density function of a standard normal distribution (i.e., probit link) and $\overset{\sim }{Y_i^1}$ has a Bernoulli distribution. The parameters from the normal linear model for the continuous outcome in equation 1 and the parameters from the probit model in equation 2 have the following relationship (see Additional file 1 Section 2 for the details):

- $$ {\beta}_0=\frac{\alpha_0-\tau }{\lambda } $$
- $$ {\beta}_j=\frac{\alpha_j}{\lambda}\cdot for\cdot j=1,\cdots, p+1. $$

When the dichotomised outcome is defined by the continuous outcome being below a known threshold, i.e., $\overset{\sim }{Y_i^2}=\mathrm{I}\left({Y}_i<\tau \right)$, the relationship of the parameters between the normal linear and probit models is:

- $$ {\beta}_0=-\left(\frac{\alpha_0-\tau }{\lambda}\right) $$
- $$ {\beta}_j=-\frac{\alpha_j}{\lambda}\cdot for\cdot j=1,\cdots, p+1. $$

The parameters of the two dichotomised outcomes (i.e., $\overset{\sim }{Y_i^1}$ and $\overset{\sim }{Y_i^2}$) differ only in the sign. Hence, for example, we can use the estimates of the normal linear model for random BG to obtain estimates of the probit model for hyperglycaemia that corresponds to random BG exceeding some threshold.

When we assumed the error terms in equation 1 are independently and identically distributed from a logistic distribution with location parameter being 0 and scale parameter being λ, we call this the logistic linear model from henceforth. The dichotomised outcomes (i.e., $\overset{\sim }{Y_i^1}$ and $\overset{\sim }{Y_i^2}$) from the logistic linear model corresponds to the logit model, and its linear predictor is represented by equation 2 and its link function is the inverse cumulative density function of a standard logistic distribution (i.e., logit link). The relationship of the parameters between the logistic linear and logit models is the same as the scenario where the error terms are normal. In summary, the scaled parameters from the normal (or logistic) linear model would correspond to the parameters from the probit (or logit) model. In particular, the scaled slope parameter of the logistic linear model will also have a log-OR interpretation. However, this interpretation does not apply to the scenario where the errors are normally distributed. Therefore, we propose to use RR to quantify the measure of association for dichotomised outcome.

### Estimation of RR

In epidemiology, effect measures for binary outcomes are usually quantified by risk difference or RR, as these measures are more intuitive and understandable [23]. In the presence of confounders, we propose to use marginal standardisation method that contrasts the risks between all individuals who are assumed to be exposed and unexposed [19]. Specifically, the risk corresponds to the marginal probability is:

3 $$ \Pr \left(\overset{\sim }{Y}=1|E=j\operatorname{}\right)=\sum \limits_{i=1}^n\Pr \left(\overset{\sim }{Y}=1|{E}_i\operatorname{}=j,{Z}_{1i}={z}_{1i},\dots, {Z}_{pi}={z}_{pi}\right){w}_i $$

where *j* takes values zero or one that corresponds to unexposed or exposed, and subscript *i* refers to the i-th individual in the study population. The marginal probability in equation 3 is a weighted average of probabilities over a target population, taking into consideration of confounders [19, 24]. Hence, using the marginal probabilities, the estimated RR is:

4 $$ \hat{\mathrm{RR}}=\frac{\hat{\Pr}\left(\overset{\sim }{Y}=1|E=1\right)}{\hat{\Pr}\left(\overset{\sim }{Y}=1|E=0\right)}=\frac{\sum_{i=1}^n\hat{\Pr}\left(\overset{\sim }{Y_i}=1|{E}_i=1,{Z}_{1i}={z}_{1i},\cdots, {Z}_{pi}={z}_{pi}\right){w}_i}{\sum_{i=1}^n\hat{\Pr}\left(\overset{\sim }{Y_i}=1|{E}_i=0,{Z}_{1i}={z}_{1i},\cdots, {Z}_{pi}={z}_{pi}\right){w}_i} $$

and ${w}_i=\frac{1}{n}$ when the target population of the standardisation is the study population [19, 24]. The quantity, $\hat{\Pr}\left(\overset{\sim }{Y_i}=1|{E}_i=j,{Z}_{1i}={z}_{1i},\cdots, {Z}_{pi}={z}_{pi}\right)$, can be estimated as follows :

5 $$ \Phi \left(\hat{\beta_0}+\hat{\beta_1}{E}_i+\sum \limits_{k=1}^p\hat{\beta_{k+1}}{Z}_{ki}\right) $$

where Φ(·) is the cumulative distribution function of standard normal (or logistic) distribution and $\hat{\beta_{\mathrm{j}}}$, for *j* = 0, ⋯, *p* + 1, are estimates from the probit (or logit) model. We can replace the estimates from probit (or logit) model with those from the normal (or logistic) linear model (i.e., $\hat{\alpha_0},\dots, \hat{\alpha_{\mathrm{p}+1}},\hat{\lambda}$). Hence, when $\overset{\sim }{Y_i^1}=\mathrm{I}\left({Y}_i>\tau \right)$, $\hat{\beta_0}=\frac{\hat{\alpha_0}-\tau }{\hat{\lambda}}$ and $\hat{\beta_{\mathrm{j}}}=\frac{\hat{\alpha_{\mathrm{j}}}}{\hat{\lambda}}$ for *j* = 1, ⋯, *p* + 1, and when $\overset{\sim }{Y_i^2}=\mathrm{I}\left({Y}_i<\tau \right)$, $\hat{\beta_0}=-\left(\frac{\hat{\alpha_0}-\tau }{\hat{\lambda}}\right)$ and $\hat{\beta_{\mathrm{j}}}=-\left(\frac{\hat{\alpha_{\mathrm{j}}}}{\hat{\lambda}}\right)$ for *j* = 1, ⋯, *p* + 1. Applying the delta method [19, 24–27] to $\hat{\mathrm{RR}}$ in equation 4, the estimated standard error (SE) of $\hat{\mathrm{RR}}$ is:

6 $$ \mathrm{se}\left(\hat{\mathrm{RR}}\right)={\left(\frac{\partial \hat{\mathrm{RR}}}{\partial \hat{\theta}}\right)}^T\hat{\mathrm{RR}}\left(\frac{\partial \hat{\mathrm{RR}}}{\partial \hat{\theta}}\right) $$

where $\hat{\theta}={\left(\hat{\alpha_0},\dots, \hat{\alpha_{\mathrm{p}+1}},\hat{\lambda}\right)}^T$ if $\hat{\mathrm{RR}}$ is estimated from linear model, and $\hat{\theta}={\left(\hat{\beta_0},\dots, \hat{\beta_{\mathrm{p}+1}}\right)}^T$ if $\hat{\mathrm{RR}}$ is estimated from probit or logit model (see Additional file 1 Section 3 for the details). The variance estimation procedure can be easily implemented in R [28].

### Simulation study

Given that the logit and probit models are two commonly used models to analyse binary outcomes, we simulated continuous outcomes data under two scenarios: logistic and normal error distributions respectively, and generated dichotomised outcomes from these data. To investigate the performance of estimates from models that are correctly specified for the continuous and dichotomised outcomes, we compared the performance of estimates from logit model applied to dichotomised outcomes with those from logistic linear model applied to continuous outcomes where the outcomes are from data with logistic error distribution. To compare estimates from probit and normal linear models when the models are correctly specified, these models are applied to data with normal error distribution. We also compared $\hat{\mathrm{RR}}$s from these correctly specified models where $\hat{\mathrm{RR}}$s were computed using the marginal standardisation method. To investigate performance of approaches that avoided dichotomisation, we compared $\hat{\mathrm{RR}}\mathrm{s}$ from marginal standardisation method that used estimates from linear model with those from the distributional approach proposed by Sauzet et al. [16]. Given that the distributional approach estimates RR by evaluating the risks at the marginal means of the linear model, it could be seen as a marginal mean method. We only considered the scenario when the data had normal error distribution in this comparison because both methods have been developed for this scenario. We called the approach proposed by Sauzet et al. [16] as *distdicho* from henceforth because it corresponded to the name of the STATA module that the authors had implemented their approach. To compute the bias and coverage probability quantities for *distdicho*, we have used the true RR value based on its definition of RR (i.e., risk of an exposed individual to an unexposed individual where both individuals have confounding profiling corresponding to the mean values of the confounders)*.*

To investigate the robustness of the models when they are applied to data with the wrong error distribution (i.e., model misspecification), we compared the performance of estimates from logit model applied to dichotomised outcomes with those from logistic linear model applied to continuous outcomes where the outcomes are from data with normal error distribution. Likewise, we compared estimates from probit and normal linear models that are applied to data with logistic error distribution. We also compared $\hat{\mathrm{RR}}\mathrm{s}$ from these mis-specified models where $\hat{\mathrm{RR}}\mathrm{s}$ were computed using the marginal standardisation method. To identify potential model misspecification in the data analysis in the real world setting, we could perform model diagnostics by testing the distribution of residuals against the assumed error distribution from the linear model and testing the appropriateness of link function from the logit or probit model with Pregibon link test [29]. To assess the distribution of residuals from the logistic linear model, the 2-sided Kolmogorov-Smirnov (KS) test is used. As KS test tends to be extremely conservative when distribution parameters are estimated from the sample, we used the Lilliefors corrected KS test [30], which is only available for normal linear model. To visualise the distribution of the residuals, we plotted the quantile-quantile plot (QQ-plot). Details of the simulation set-up are provided in the next paragraph.

We simulated a continuous outcome as a function of a binary exposure variable, *E*, and two binary confounders, *Z*_1_ and *Z*_2_. We first simulated *Z*_1_ and *Z*_2_ from Bernoulli trials with success probabilities being 0.4 and 0.6 respectively, and then simulated *E* from a Bernoulli trial with success probability $\Pr \left(E=1|{Z}_1,{Z}_2\right)=0.34\times \exp \left\{\ln \left(\sqrt{2}\right){Z}_1+\ln \left(\sqrt{2}\right){Z}_2\right\}.$ We simulated the continuous outcome based on the following:

7 $$ {Y}_i={\alpha}_0+{\alpha}_1{E}_i+{\alpha}_2\left({Z}_{1i}-0.4\right)+{\alpha}_3\left({Z}_{2i}-0.6\right)+{\varepsilon}_{\mathrm{i}} $$

where ε_i_ had a normal (or logistic) distribution with sample size set to 1000. We set α_0_ = 0.4 and α_2_ = α_3_ = 0.5, and considered the following α_1_ values that reflected increasing negative exposure effect on the outcome: 0, −0.15 and − 0.3. We also considered the following λ values: 1, 2 and 0.5. We considered the dichotomised outcome corresponding to *Y*_*i*_ s exceeding a pre-specified threshold, $\overset{\sim }{Y_i}=\mathrm{I}\left({Y}_i>\tau \right)$. To vary the baseline risk, we used threshold values corresponding to the 7.5-th, 15-th, 30-th, 50-th, 70-th, 85-th, and 92.5-th percentiles of *Y*_*i*_ = α_0_ + ε_i_ which represents the outcome of an individual with the mean outcome corresponding to the marginal mean where all subjects in the population are assumed to be unexposed. We conducted 1000 simulation iterations to assess the bias, SE, coverage probability (i.e., the probability that the constructed 95% confidence interval contains the true value of the parameter), type 1 error, and power of both $\hat{\beta_1}$ and $\hat{\mathrm{RR}}$ from normal linear, logistic linear, probit and logit models. We also assessed the performance of the KS test, Lilliefors corrected KS test and Pregibon link test for model misspecification with the type 1 error and power of these tests.

### Empirical example

The real dataset consists of the BG readings from index inpatient admissions of patients with type 2 diabetes mellitus admitted within the pre- and post- 60 days of a pilot intervention program designed to improve inpatient management of hyperglycaemia in selected wards of a tertiary hospital in Singapore in 2013. Each inpatient admission has BG readings at multiple time-points. To assess whether the intervention program could reduce the proportion of admissions with at least one hyperglycaemia event, we performed the normal and logistic linear models on the log-transformed maximum BG levels within an admission, with adjustment for age, gender, ethnicity, and emergency admission status. We used the same plots and tests in the simulation study to assess the distributional assumption of the error term in the linear model. Four common thresholds in inpatient management of hyperglycaemia with increasing severity: 10, 14, 16, and 20 mmol/L [31–33], were used in the analyses. We also performed the probit and logit models on the dichotomised outcomes at each threshold and assessed the appropriateness of the links using Pregibon link test. We reported 2-sided *P* values for the association between intervention and hyperglycaemia risk.

We used R version 3.3.2 (Vienna, Austria) to perform the simulation and analyse the real dataset [28]. To perform the normal and logistic linear models, we used the R package, gamlss (Generalised Additive Models for Location Scale and Shape) [34]. To obtain $\hat{\mathrm{RR}}$ from the distributional approach [16] (*distdicho*), we implemented their method in R.

## Results

### Simulation results

#### *β*_1_ estimate ($\hat{\beta_1}$) from simulation

**Fig. 1:**
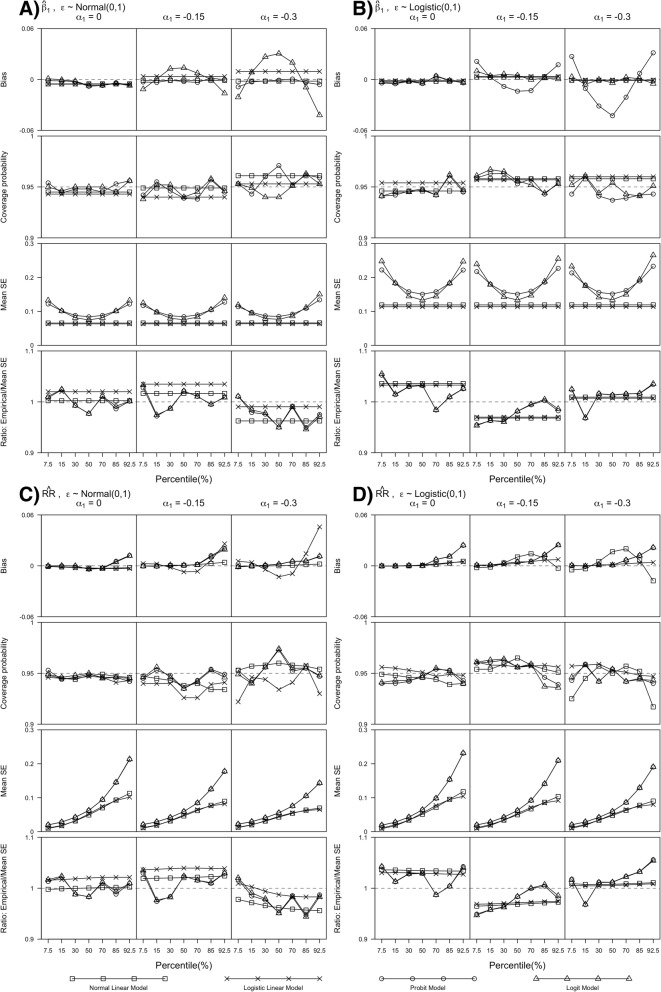
Simulations results for bias, coverage probability and standard error for both $\hat{\beta_1}$ and $\hat{\mathrm{RR}}$. Panel (**a**) and (**b**) plot the simulation results for $\hat{\beta_1}$ and panel (**c**) and (**d**) plot the simulation results for $\hat{\mathrm{RR}}$, when errors are normally (or logistically) distributed with mean (or location) 0 and standard deviation (or scale) 1, and α_1_ = 0,−0.15, and −0.3. Dashed lines are 0, 0.95 and 1 for Bias, Coverage probability and Ratio: Mean/Empirical standard error (SE) respectively, which correspond to no bias, 95% coverage probability and mean and empirical SEs are the same. Normal and logistic linear models mean linear model with the error terms assumed to have normal and logistic distribution respectively

β_1_ is the slope parameter associated with exposure for probit and logit model, and it corresponds to the scaled slope parameter associated with exposure from the linear models. Regardless of the threshold values and exposure effect sizes, we found, in general, $\hat{\beta_1}$s from normal linear and probit models had biases close to 0 and coverage probabilities close to 95%, which suggests comparable performance between these two models in terms of unbiasedness and coverage probabilities, when outcomes had standard normal errors (see Fig. 1a). Similar findings were observed for logistic linear and logit models when outcomes had standard logistic errors (see Fig. 1b). Model misspecification from applying logistic (or normal) linear model to continuous outcomes with standard normal (or logistic) errors gave unbiased estimates with coverage probabilities close to 95% across different threshold values. However, the mis-specified binary models produced biased estimates especially when the effect size was large (β_1_ =  − 0.3).

**Fig. 2:**
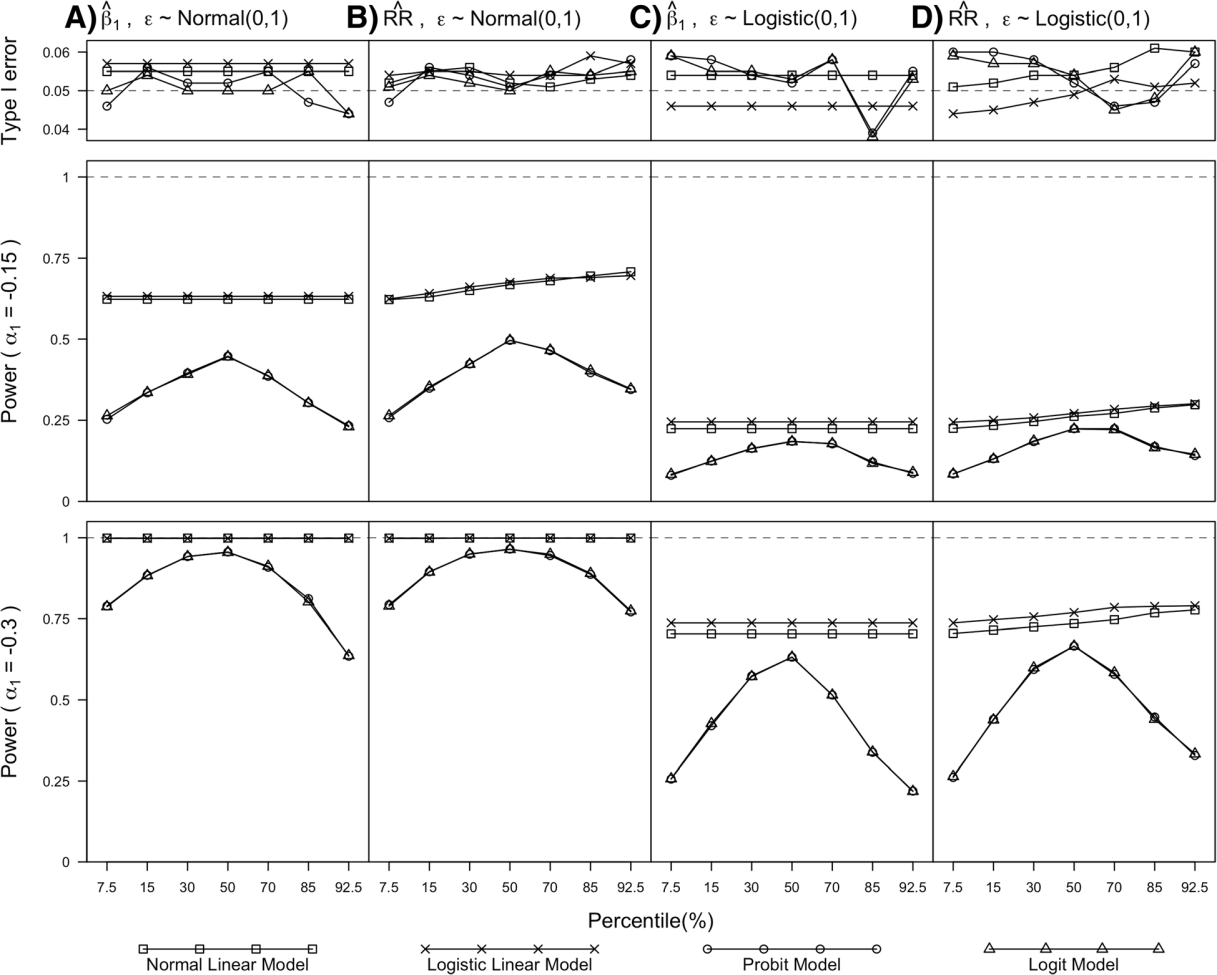
Simulation results for type 1 error and power when α_1_ =  − 0.15 and α_1_ =  − 0.3. Panel (**a**) and (**c**) plot the simulation results for $\hat{\beta_1}$ and panel (**b**) and (**d**) plot the simulation results for $\hat{\mathrm{RR}}$. Dashed lines are plotted at 0.05 and 1 for Type 1 error and Power, corresponding to 0.05 type 1 error and 100% power respectively. Normal and logistic linear model means linear model with the error terms assumed to have normal and logistic distribution respectively

The SEs of $\hat{\beta_1}$ from the normal and logistic linear models were comparable but consistently smaller when compared to probit and logit models where the differences were more pronounced when threshold values deviated from 50-th percentile threshold value, regardless of exposure effect sizes and error distributions. Empirical and mean SEs of $\hat{\beta_1}$ for each model were comparable, regardless of exposure effect sizes, threshold values and error distributions. Although the type 1 errors of $\hat{\beta_1}$ were close to 0.05 for all models, normal and logistic linear models had higher power than probit and logit models respectively with differences being more pronounced when threshold values were at 7.5-th and 92.5-th percentiles (see Fig. 2a and c). Similar findings were observed when λ = 0.5, 2 (see Additional file 1 Section 4, Additional file 1: Figures S1 to S4).

#### Relative risk estimate ($\hat{RR}$) from simulation

Similar to $\hat{\beta_1}$s, in general, we found $\hat{\mathrm{RR}}$s from all four models unbiased with coverage probabilities close to 95% across exposure effect sizes and threshold values when outcomes had standard normal (or logistic) error distribution (see Fig. 1c and d). Except when threshold values were at 92.5-th percentile, the estimates from mis-specified linear models had somewhat pronounced biases, but within 10% of the true value. Similar to $\hat{\beta_1}$s, the SEs of $\hat{\mathrm{RR}}$ from the normal and logistic linear models were smaller than the probit and logit models. However, the differences increased as threshold values increased from 7.5-th to 92.5-th percentile. $\hat{\mathrm{RR}}$ had similar findings as $\hat{\beta_1}$ for ratio between empirical and mean SEs, type 1 error and power. Similar findings for $\hat{\mathrm{RR}}$ were observed when λ = 0.5, 2 (see Additional file 1 Section 4, Additional file 1: Figures S1 to S4). When comparing approaches that avoided dichotomisation, we found $\hat{\mathrm{RR}}$ from the marginal standardisation method, in general, had comparable performance as the distributional approach (*distdicho*) in terms of bias, coverage, empirical SE over mean SE, type 1 error and power (see Additional file 1 Section 4, Additional file 1: Figures S5 to S7) where the bias and coverage quantities were based on the true RR value as defined by the marginal mean method. The mean SEs of *distdicho* were only slightly larger than the SE of the marginal standardisation method when the threshold values and exposure effect sizes were large.

#### Model diagnostics

**Fig. 3:**
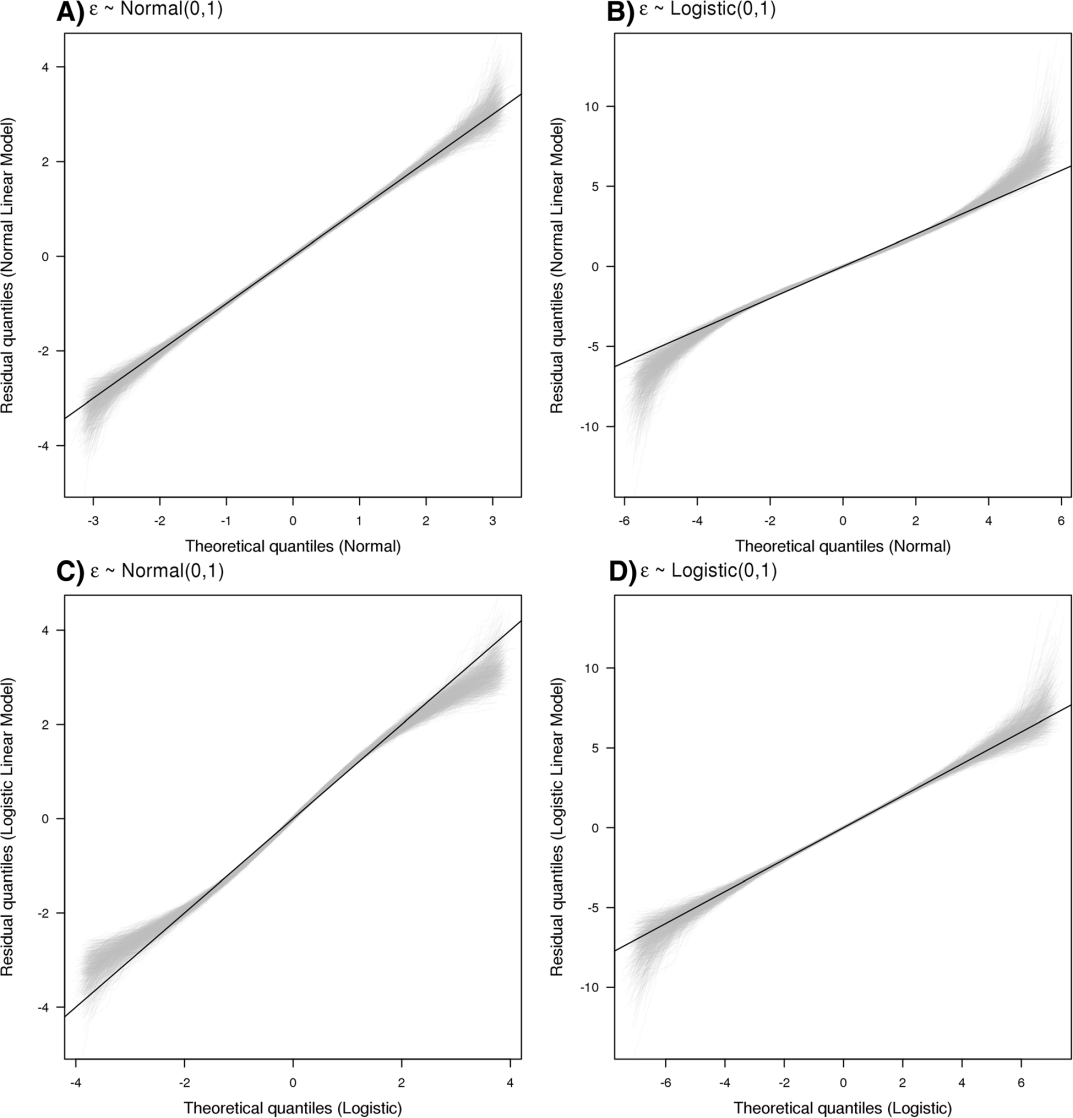
Quantile-quantile plot of residuals from linear models applied to simulated datasets when α_1_ = 0. Panel (**a**) and (**b**) plot the residuals from linear models with normal errors. Panel (**c**) and (**d**) plot the residuals from linear models with logistic errors. Gray lines plot quantile-quantile lines for 1000 simulations and black line is the 45-degree line. Normal and logistic linear models mean linear model with the error terms assumed to have normal and logistic distribution respectively

Model diagnostics could alleviate the bias due to model misspecification in the error distribution as observed in the previous simulation findings. When the effect size and scale parameter were zero and one respectively, the QQ plot of residuals from linear models followed the 45-degree line when applied appropriately (see Fig. 3a and d). Pregibon link tests applied on binary models had type 1 errors close to 0.05 with power lower than 5%. However, KS test for logistic linear model and Lilliefors corrected KS test for normal linear model had type 1 error much lower than 0.05 with power close to 5% and type 1 error close to 0.05 with power close to 80% respectively (see Table 1). Hence, the diagnostic test for linear model is more powerful than the one for probit and logit models. Similar findings were observed across different effect sizes and scale values (results not presented).

**Table 1:** Simulation results for model diagnostics when α_1_ = 0

		ε~Normal(0, 1)	ε~Logistic(0, 1)
Model	Percentiles	Proportion of rejection at 5% significance level	Proportion of rejection at 5% significance level
Normal Linear Model ^a^	NA ^c^	0.053	0.822
Probit Model ^b^	0.075	0.037	0.043
	0.15	0.039	0.032
	0.3	0.041	0.042
	0.5	0.033	0.038
	0.7	0.028	0.039
	0.85	0.04	0.044
	0.925	0.038	0.029
Logistic Linear Model ^a^	NA ^c^	0.052	0
Logit Model ^b^	0.075	0.039	0.032
	0.15	0.036	0.039
	0.3	0.036	0.038
	0.5	0.038	0.034
	0.7	0.034	0.034
	0.85	0.042	0.052
	0.925	0.028	0.041

^a^Lilliefors corrected Kolmogorov-Smirnov test were used to test whether residuals from normal linear model had a normal distribution, and Kolmogorov-Smirnov test was used to test whether residuals from logistic linear models had a logistic distribution. ^**b**^Pregibon link test was used to test whether probit or logit link was appropriate. cThe same normal (or logistic) linear model is applied across different threshold values.

### Empirical results

**Fig. 4:**
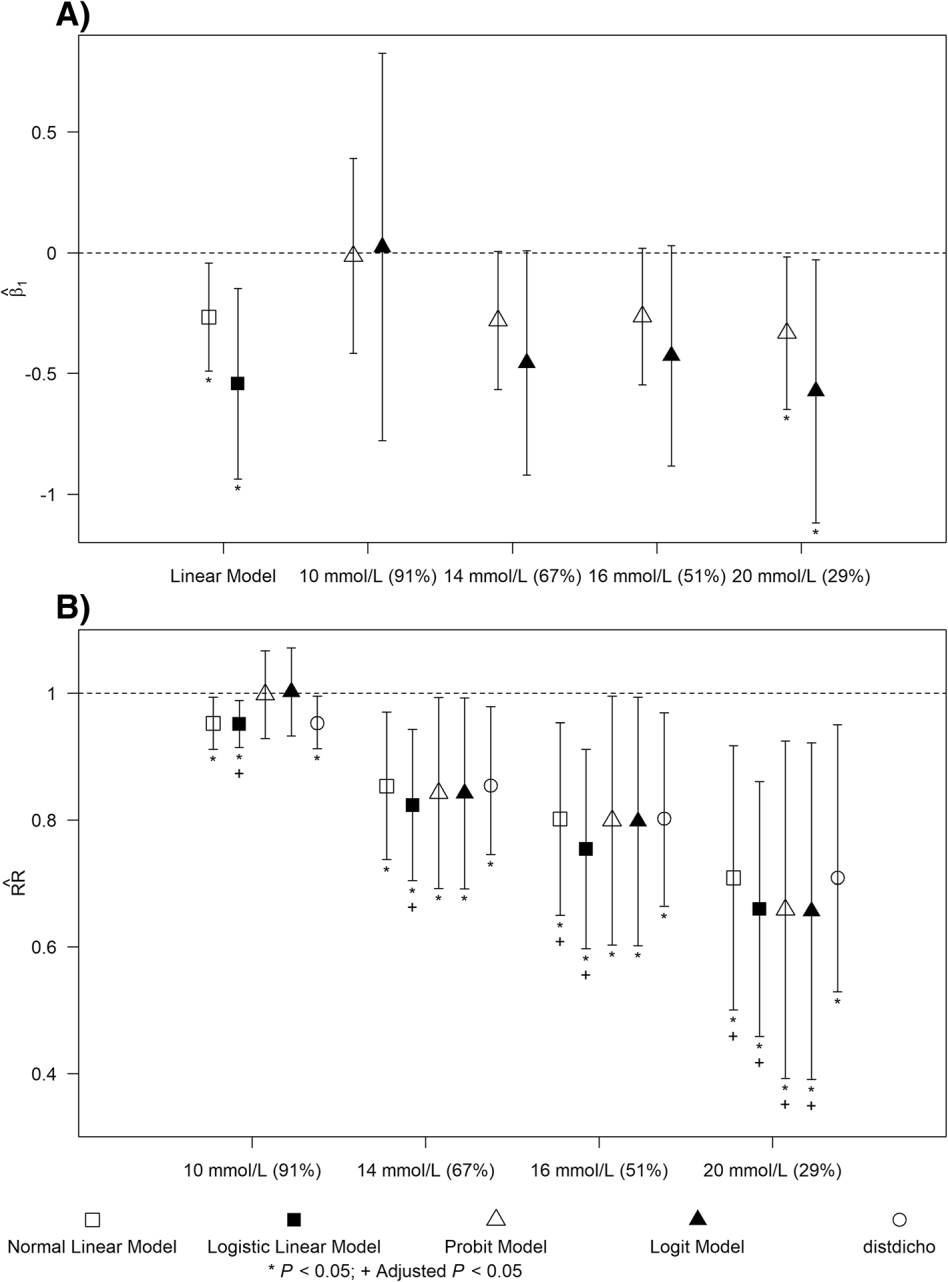
Effect of intervention on risk of hyperglycaemia with respect to different degree of severity. Panel (**a**) and (**b**) plot $\hat{\beta_1}$ s and $\hat{\mathrm{RR}}$ s with their corresponding 95% confidence intervals (i.e., vertical lines) for linear, probit and logit models respectively. * indicates a *P*-value less than 0.05 without adjustment for multiple testing. + indicates a *P*-value less than 0.05 after adjustment for multiple testing

We obtained the index inpatient admissions of 317 patients with type 2 diabetes mellitus, where 175 and 142 admissions occurred before and after intervention respectively. The patients were generally elderly where the mean age before intervention is 70 (SD = 14) years and the mean age after intervention is 67 (SD = 16) years. Majority of the patients were females (before: 55% vs after: 61%), and the majority were Chinese (before: 60% vs after: 56%), followed by Malay (before: 21% vs after: 21%), Indian (before: 13% vs after: 19%), and Others (before: 6% vs after: 4%). The average of maximum BG within an admission is 15.9 (SD = 6) mmol/L after intervention and it was significantly lower when compared with the average of maximum BG before intervention (i.e., mean of maximum BG = 17.3; SD = 6 mmol/L) without adjustment for confounders. To assess whether the proportion of admissions with at least one hyperglycaemia event was reduced after the intervention and to avoid dichotomisation, we performed the normal and logistic linear models on the log-transformed maximum BG within an admission with adjustment for confounders. For an admission to have at least one hypoglycaemia event within an admission, its maximum BG has to exceed a threshold value, such as: 10 mmol/L (before: 91% vs after: 92%), 14 mmol/L (before: 67% vs after: 57%), 16 mmol/L (before: 51% vs after: 41%), and 20 mmol/L (before: 29% vs after: 20%). Figure 4a compares the adjusted $\hat{\beta_1}$s which were associated with intervention indicator in the linear predictor of the linear, probit and logit models. For all thresholds except 10 mmol/L, we found estimates from normal linear and probit models similar, and estimates from logistic linear and logit models similar. The 95% confidence intervals of $\hat{\beta_1}$s from linear models were narrower than probit and logit models, while the confidence intervals among probit and logit models were wider when threshold values corresponded to baseline risks further from 50-th percentile (i.e., 29 and 91%). The linear models indicated that $\hat{\beta_1}$ was significantly different from zero, but the probit and logit models had the same conclusion only when the threshold value was 20 mmol/L.

Although $\hat{\mathrm{RRs}}$ were similar across all approaches within each threshold except at 10 mmol/L, the confidence intervals of $\hat{\mathrm{RR}}$s computed from normal and logistic linear models by using marginal standardisation method, and normal linear model by using marginal mean method (*distdicho*) were narrower than those computed from probit and logit models by using marginal standardisation method (see Fig. 4b). The confidence intervals of $\hat{\mathrm{RR}}$s from all approaches were increasing as the baseline risks decreased. Probit and logit models indicated that $\hat{\mathrm{RRs}}$ were significantly different from one for all thresholds except 10 mmol/L, whereas marginal standardisation and mean methods applied to linear models also indicated significant difference from one when the threshold value was 10 mmol/L. To account for multiple testing because four thresholds were used to define hyperglycaemia, we adjusted the *P* values for $\hat{\mathrm{RR}}$ using Bonferroni correction [35]. After correction, the marginal standardisation method suggested significant difference in risks for all four thresholds with the logistic linear model and 16 and 20 mmol/L with the normal linear model where the remaining two thresholds had borderline significance (i.e., adjusted *P* values between 0.05 and 0.1). All thresholds except 10 mmol/L had borderline significance for the marginal mean method applied to normal linear model (*distdicho*) with adjusted *P* values ranging from 0.088 to 0.108. Significant difference in risk was observed for only one threshold (i.e., 20 mmol/L) with no borderline significance for the remaining three thresholds when marginal standardisation method was applied to probit and logit models. Similarly, after multiple testing adjustment for $\hat{\beta_1}$ from probit and logit models, the results were insignificant with no borderline significance for all thresholds.

**Fig. 5:**
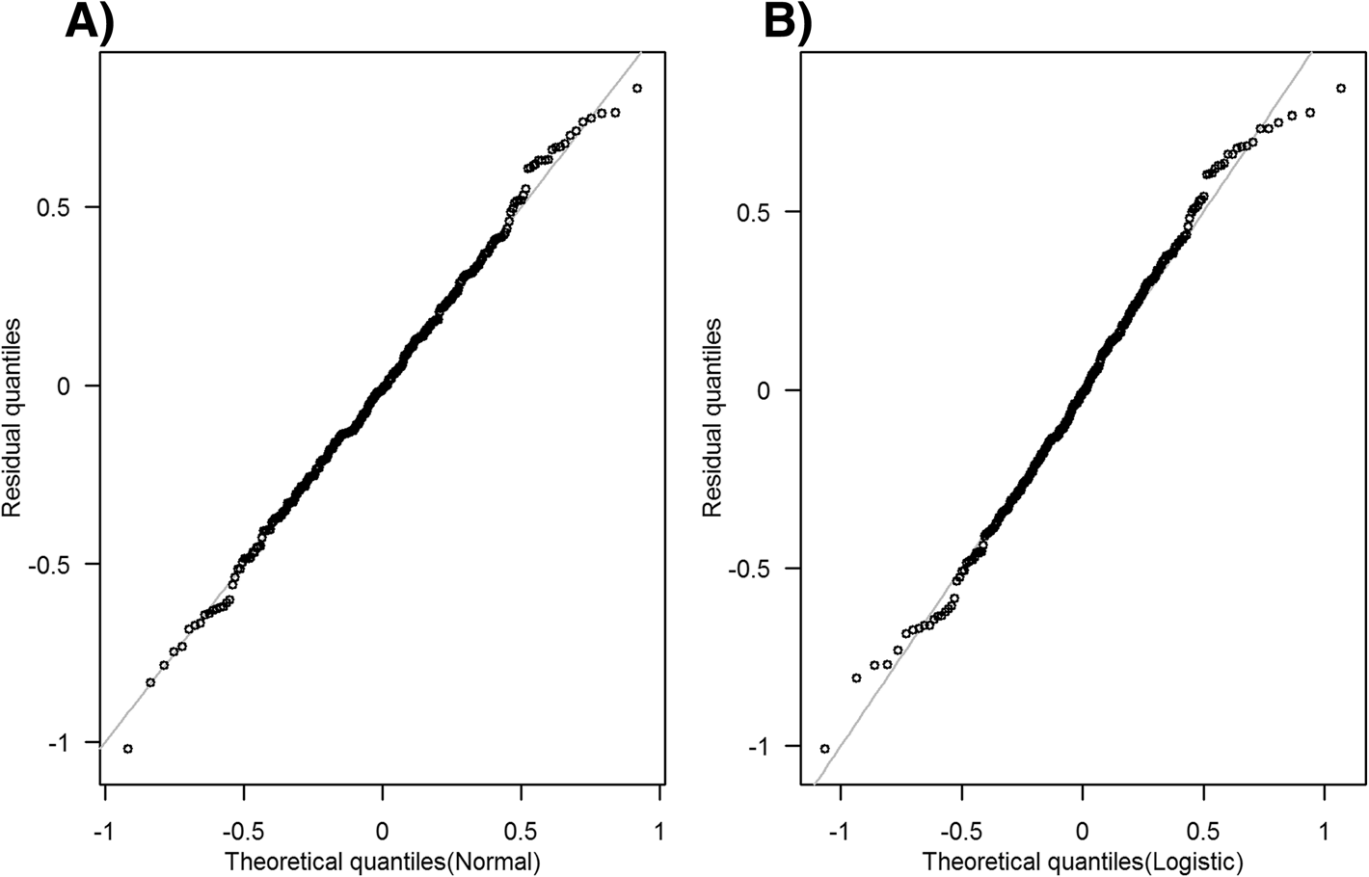
Quantile-quantile plot of residuals from linear models applied to real dataset. Black line is the 45-degree line. Panel (**a**) plots the residuals from linear model with normal errors. Panel (**b**) plots the residuals from linear model with logistic errors

The QQ plot of residuals from normal and logistic linear models suggested that the distributional assumptions were reasonable (see Fig. 5), with Lilliefors corrected KS test and KS test *P* values being 0.91 and 0.80 for normal and logistic linear models respectively. Similar findings were observed with binary models, where the Pregibon link test results showed insignificant results for all thresholds except 20 mmol/L for Probit only (*P* value = 0.04), which became insignificant after Bonferroni correction.

## Discussion

Dichotomisation of continuous outcome is a common and appealing analytical approach especially when the dichotomisation process is also practiced in the clinical setting. However, the use of dichotomisation has been greatly criticised for non-negligible loss of power and increased variability in the estimate [6–10]. In particular, the magnitude of loss in power is greater when the threshold value is distant from the mean or median [7, 9], which corresponds to the scenario where OR approximates RR. To avoid the drawbacks of dichotomisation and facilitate the interpretation of binary representations of continuous outcomes from linear models, we proposed to transform estimates from linear models to RR through marginal standardisation. We evaluated the performance of our proposed approach, and compared it with the dichotomized and distributional approaches using both simulated and real datasets.

When comparing marginal standardisation method that avoided and did not avoid dichotomisation, our simulation results suggested β_1_ and RR estimates from linear, probit and logit models were generally unbiased when applied appropriately, but probit and logit models had larger SEs and smaller power than those estimates from linear models. The improvement in precision and power of estimates from continuous outcomes were also reported in other studies [11, 12, 16, 36]. Although β_1_ estimates from mis-specified binary models had somewhat pronounced biases when effect size was large, past studies have found that small sample size or extremely common (or rare) binary outcomes could lead to biased estimates from logit model, which were negligible when compared to the magnitude of the SEs [37]. The OR approximates to the RR when baseline risk is low (i.e., threshold at 92.5-th percentile), however, the reduction in precision and power of OR estimates from logistic linear model to logit model was also more pronounced when baseline risk is low.

Often, model diagnostics are performed to assess model misspecification [38]. Our simulation results suggested that the Lilliefors corrected KS test and KS test had better power in assessing the distributional assumptions of the error terms for linear models when compared to Pregibon link tests for binary models. Our findings were consistent with a previous study that reported low power when differentiating probit and logit links in binary models [39]. These findings were expected as probit and logit links are known to be similar [40].

From the real data analyses, in general, we found the estimates from normal linear and probit models to be similar, and this phenomenon was also observed in estimates from logistic linear and logit models. However, estimates from probit and logit models had wider confidence intervals and fewer significant findings than those from linear models. These observations were consistent with findings from our simulation and the literature [11, 12, 15]. Although we had used the log-transformed outcome in the real dataset analyses, $\hat{\mathrm{RR}}\mathrm{s}$ obtained from this transformation is equivalent to that obtained from the original outcome without transformation, which corresponds to a multiplicative regression model with log-normal [41] or log-logistic error distribution [42]. When comparing approaches that avoided dichotomisation within normal linear model in the real data analysis, we found the marginal standardisation and marginal mean methods had similar $\hat{\mathrm{RR}}\mathrm{s}$. From the Jensen’s inequality [43], the estimated probability obtained via marginal mean method could differ from that obtained via marginal standardisation method because the link function is not linear. However, if the range of the linear predictor values is in a neighbourhood where a linear function can be used to approximate the link function, $\hat{\mathrm{RR}}\mathrm{s}$ from the two methods can be similar. Although these two methods gave similar results in our real data analysis, the marginal standardisation method is more commonly used in epidemiology studies [19, 25–27, 44] when compared with the marginal means method, and can be viewed as a special case of G-computation method in the causal inference literature [19], and can be generalised to binomial models with other link functions [26].

When faced with multiple threshold values to dichotomise the continuous outcome, the conventional approach would apply probit or logit model to the dichotomised outcome at each threshold value resulting in a multiple testing problem when assessing whether β_1_ (or RR) equals to 0 (or 1). In the real dataset analyses, after applying Bonferroni correction to account for the multiple testing problem with binary models, only RR defined at threshold value corresponding to 20 mmol/L was significant while β_1_s for all threshold values were not significant. However, with linear models, we avoided the multiple testing problem for β_1_. We first assessed whether β_1_ returned a significant finding before proceeding to identify the threshold value where RR ≠ 1. Linear models in the real dataset analyses returned significant findings for β_1_ and the follow-up analyses for RR, in general, returned significant results at various threshold values after accounting for multiple testing. In particular, applying marginal standardisation method to normal linear model to obtain $\hat{\mathrm{RR}}\mathrm{s}$ provided stronger evidence of reduction in hyperglycaemia risk after intervention although $\hat{\mathrm{RR}}\mathrm{s}$ were similar across methods. For future studies involving multiple thresholds, one could mimic the ANOVA testing procedure by starting with a test for β_1_ from the linear model before proceeding to perform post-hoc tests for RR at each threshold value with marginal standardisation or mean method.

Our proposed method has some limitations. We did not consider dichotomisation based on two thresholds, e.g., I(τ_1_ < *Y*_*i*_ < τ_2_) or I(*Y*_*i*_ < τ_1_, *Y*_*i*_ > τ_2_), but our proposed approach can be extended to this scenario and it is beyond the scope of this paper. Although we have presented an application to binary exposure, our approach can be applied to categorical or continuous exposure as well [11, 16, 36, 45]. The marginal standardisation and mean methods in this paper were used to estimate RR only, but both methods can also be used to estimate absolute risk reduction, RR reduction and number needed to treat pending on the research question [44, 46].

## Conclusions

In conclusion, we have extended the marginal standardised method that estimated RR using estimates from the linear model when the continuous outcome has a binary representation corresponding to the outcome exceeding or being below some pre-specified threshold value. By avoiding the application of probit or logit model on the dichotomised outcome, we obtained an unbiased RR estimate that was more precise and powerful with marginal standardisation and mean methods, and a more powerful model diagnostic test that could potentially alleviate potential issues associated with model misspecification in the error distribution. We provided guidance for future analyses involving dichotomised outcomes to facilitate interpretation, including settings involving multiple thresholds for the same continuous outcome.

## Additional file

Additional file 1: Additional theoretical proofs and additional simulation results. The file consists of four sections. **Section 1.** Comparison of marginal standardisation approach to marginal means approach **Section 2.** Derivation details for the relationship between parameters from linear models to probit or logit models **Section 3.** Variance of estimates **Section 4.** Additional simulation results (DOCX 2100 kb)

## Data Availability

The computer code supporting the conclusions of this article is available in: http://blog.nus.edu.sg/dasa/transoutcome/

## Abbreviations

ANOVA: Analysis of Variance
BG: Blood Glucose
KS: Kolmogorov-Smirnov
OR: Odds Ratio
QQ-plot: Quantile-Quantile plot
RR: Relative Risk
SD: Standard Deviation
SE: Standard Error
