# Supplementary material for: Using marginal standardisation to estimate relative risk without dichotomising continuous outcomes
Source: BMC Med Res Methodol. 2019 Jul 29;19:165. doi: 10.1186/s12874-019-0778-9 (PMC6664591; doi:10.1186/s12874-019-0778-9)
Supplement: Supplementary file 1 — Additional theoretical proofs and additional simulation results. The file consists of four sections. Section 1. Comparison of marginal standardisation approach to marginal means approach Section 2. Derivation details for the relationship between parameters from linear models to probit or logit models Section 3. Variance of estimates Section 4. Additional simulation results (DOCX 2100 kb) [file 12874_2019_778_MOESM1_ESM.docx]

**SECTION 1: COMPARISON OF MARGINAL STANDARDISATION APPROACH TO MARGINAL MEANS APPROACH**

**One-sample case**

Let $Y_{i}$ be the observed outcome for the $i$-th individual, where $i=1,...,n$. Below is the linear model for a continuous outcome under the one-sample setting:

$$Y_{i}=\alpha_{0}+\varepsilon_{i},(A1.1)$$

where the error terms are assumed to be independent and identically distributed from a normal distribution with mean being 0 and standard deviation being $\lambda$, i.e., $\varepsilon_{i} \sim\mathrm{Normal}(0,\lambda)$ for $i=1,...,n$.

Following the definition of marginal standardisation with equal weights for population, risk for the true outcome being greater than $\tau$ (a pre-specified threshold) could be represented as

$$R=\frac{1}{n}\sum_{i=1}^{n} \Pr(Y_{i}>\tau)=\Phi(\frac{\alpha_{0}-\tau}{\lambda}),(A1.2)$$

where $\Phi\left( \cdot\right)$ represents the cumulative distribution function of standard normal distribution. By replacing $\alpha_{0}$ and $\lambda$ with their maximum likelihood estimators (MLE) $\hat{\alpha_{0}} =\overline{Y}$ and $\hat{\lambda}=\sqrt{\frac{{\sum_{i=1}^{n} (Y_{i}-\overline{Y})}^{2}}{n}}$, we could estimate $R$ as,

$$r=\Phi(\frac{\hat{\alpha_{0}}-\tau}{\hat{\lambda}}).(A1.3)$$

Therefore, equation A1.3 is equivalent to the one-sample risk formula using marginal means as described by Suissa ^1^,

$$r=\Pr\left( Z>c^{*} \right)=1-\Phi\left( \frac{\tau-\overline{Y}}{s} \right)=\Phi\left( \frac{\overline{Y}-\tau}{s} \right),(A1.4)$$

where $\overline{Y}$ and $s$are also the MLEs of sample mean and sample standard deviation, which is slightly different from Peacock et al’s approach ^2^, where s is a method of moments estimator of standard deviation with denominator being n-1 instead of n.

**Two-sample case**

Let $E_{i}$ be the exposure variable, which takes a value of 1 if the $i$-th individual is exposed to the exposure variable, and 0 otherwise. The relationship between the exposure and outcome could be modelled as follows:

$$Y_{i}=\alpha_{0}+\alpha_{1}E_{i}+\varepsilon_{i},(A1.5)$$

where the error terms are assumed to be independent and identically distributed from a normal distribution with mean being 0 and standard deviation being $\lambda$, i.e., $\varepsilon_{i} \sim\mathrm{Normal}(0,\lambda)$ for $i=1,...,n$. Using marginal standardisation and following the derivation for the one-sample case, when there are no confounders, the relative risk (RR) would be represented as follows

$$\mathrm{RR}=\frac{\Phi\left( \frac{\alpha_{0}+\alpha_{1}-\tau}{\lambda} \right)}{\Phi\left( \frac{\alpha_{0}-\tau}{\lambda} \right)},(A1.6)$$

Similarly, if we are using MLE estimates from the linear model to replace the true parameter, then we could obtain the estimated RR as follows

$$\hat{\mathrm{RR}}=\frac{\Phi\left( \frac{\hat{\alpha_{0}}+\hat{\alpha_{1}}-\tau}{\hat{\lambda}} \right)}{\Phi\left( \frac{\hat{\alpha_{0}}-\tau}{\hat{\lambda}} \right)},(A1.7)$$

Let $\hat{Y_{1}}$and $\hat{Y_{0}}$ denote the sample mean of the outcomes for individuals being exposed and unexposed respectively. Hence:

$$\hat{Y_{0}}=\hat{\alpha_{0}},$$

$$\hat{Y_{1}}=\hat{\alpha_{0}}+\hat{\alpha_{1}}$$

Equation A1.6 could be represented as

$$\hat{\mathrm{RR}}=\frac{\Phi\left( \frac{\hat{Y_{1}}-\tau}{\hat{\lambda}} \right)}{\Phi\left( \frac{\hat{Y_{0}}-\tau}{\hat{\lambda}} \right)},(A1.8)$$

where $\hat{\lambda}$ is the maximum likelihood estimate. The $\hat{\mathrm{RR}}$ proposed by Peacock et al that is based on the marginal means approach is:

$$\hat{\mathrm{RR}}=\frac{\Phi\left( \frac{\hat{Y_{1}}-\tau}{s} \right)}{\Phi\left( \frac{\hat{Y_{0}}-\tau}{s} \right)},(A1.9)$$

where $s$ is the sample standard deviation, whereas, Suissa’s approach gives

$$\hat{\mathrm{RR}}=\frac{\Phi\left( \frac{\hat{Y_{1}}-\tau}{s_{1}} \right)}{\Phi\left( \frac{\hat{Y_{0}}-\tau}{s_{0}} \right)},(A1.10)$$

where $s_{1}$ and $s_{0}$ are the maximum likelihood estimates for the standard deviation of samples for exposed and unexposed groups respectively. Therefore, we could see that the approach proposed by Peacock et al and Suissa differ from our approach in terms of the estimator for standard deviation.

**References**

1. Suissa S. Binary methods for continuous outcomes: a parametric alternative. *J Clin Epidemiol* 1991;**44**(3):241-8.

2. Peacock J, Sauzet O, Ewings S, Kerry S. Dichotomising continuous data while retaining statistical power using a distributional approach. *Statistics in medicine* 2012;**31**(26):3089-3103.

**SECTION 2: DERIVATION DETAILS FOR THE RELATIONSHIP BETWEEN PARAMETERS FROM LINEAR MODELS TO PROBIT OR LOGIT MODELS**

Letting $Y_{i}$, $E_{i}$ and $Z_{1i},\cdots,Z_{pi}$ be the outcome, exposure and confounders of the i-th individual respectively, the linear model for a continuous outcome can be expressed as follows:

$$Y_{i}=\alpha_{0}+\alpha_{1}E_{i}+\sum_{k=1}^{p} \alpha_{k+1}Z_{ki}+\varepsilon_{i},(A2.1)$$

where the error terms are assumed to be independent and identically distributed from a normal distribution with mean being 0 and standard deviation being $\lambda$, i.e., $\varepsilon_{i} \sim\mathrm{Normal}(0,\lambda)$ for $i=1,...,n$.

Suppose the dichotomised outcome is defined by the continuous outcome exceeding a known threshold, i.e.,$\overset{̃}{Y_{i}^{1}}=I(Y_{i}>\tau)$, where $\tau$ is the threshold and $I\left( \cdot\right)$ is the indicator function. Following equation A2.1, the probability of the dichotomised outcome for the i-th individual is:

$$\Pr\left( \overset{̃}{Y_{i}^{1}}=1|E_{i},Z_{1i},\cdots,Z_{pi} \right)=\Pr\left( Y_{i}>\tau|E_{i},Z_{1i},\cdots,Z_{pi} \right)$$

$$=\Pr\left( \varepsilon_{i}> \tau-\left( \alpha_{0}+\alpha_{1}E_{i}+\sum_{k=1}^{p} \alpha_{k+1}Z_{ki} \right) \right)$$

(Because of the symmetric property of normal distribution)

$$=\Pr\left( \varepsilon_{i}< \left( \alpha_{0}-\tau+\alpha_{1}E_{i}+\sum_{k=1}^{p} \alpha_{k+1}Z_{ki} \right) \right)$$

$$=\Pr\left( \frac{\varepsilon_{i}}{\lambda}< \frac{\left( \alpha_{0}-\tau+\alpha_{1}E_{i}+\sum_{k=1}^{p} \alpha_{k+1}Z_{ki} \right)}{\lambda} \right)$$

$$=\Phi\left( \frac{\alpha_{0}-\tau+\alpha_{1}E_{i}+\sum_{k=1}^{p} \alpha_{k+1}Z_{ki}}{\lambda} \right), (A2.2)$$

where $\Phi\left( \cdot\right)$ represents the cumulative distribution function of standard normal distribution. Equation A2.2 also corresponds to the probit model of the binary outcome, $\overset{̃}{Y_{i}^{1}}$. Therefore, the intercept (i.e., $\beta_{0}$) and slopes (i.e., $\beta_{1},\cdots, \beta_{p+1}$) of the probit model equal to $\frac{\alpha_{0}-\tau}{\lambda}$ and $\frac{\alpha_{j}}{\lambda}$ for $j=1,\cdots, p+1$ respectively.

In the case where the dichotomised outcome is defined by the continuous outcome falling below a known threshold, i.e., $\overset{̃}{Y_{i}^{2}}=I\left( Y_{i}<\tau\right)$, the probability of the dichotomised outcome is:

$$\Pr\left( \overset{̃}{Y_{i}^{2}}=1|E_{i},Z_{1i},\cdots,Z_{pi} \right)=\Pr\left( Y_{i}<\tau|E_{i},Z_{1i},\cdots,Z_{pi} \right)$$

$$=\Pr\left( \varepsilon_{i}< \tau-\left( \alpha_{0}+\alpha_{1}E_{i}+\sum_{k=1}^{p} \alpha_{k+1}Z_{ki} \right) \right)$$

$$=\Pr\left( \frac{\varepsilon_{i}}{\lambda}< -\frac{\left( \alpha_{0}-\tau+\alpha_{1}E_{i}+\sum_{k=1}^{p} \alpha_{k+1}Z_{ki} \right)}{\lambda} \right)$$

$$=\Phi\left( -\frac{\alpha_{0}-\tau+\alpha_{1}E_{i}+\sum_{k=1}^{p} \alpha_{k+1}Z_{ki}}{\lambda} \right). (A2.3)$$

The intercept and slopes from the probit model in equation A2.3 equal to $\beta_{0}=-(\frac{\alpha_{0}-\tau}{\lambda})$ and $\beta_{j}=-(\frac{\alpha_{j}}{\lambda})$ for $j=1,\cdots,p+1$, where the difference with those from equation A2.2 lies in the sign.

If the error terms are assumed to follow a logistic distribution with location parameter being 0 and scale parameter being $\lambda$, i.e., $\varepsilon_{i} \sim Logistic(0,\lambda)$ for $i=1,...,n$, the same relationship follows immediately, with $\Phi\left( \cdot\right)$ representing the cumulative distribution function of standard logistic distribution.

**SECTION 3: VARIANCE OF ESTIMATES**

Let $Y_{i}$, $E_{i}$ and $Z_{ki}, k=1,\ldots,p,$ be the observed outcome, exposure and confounders for the i-th individual respectively, where $i=1,...,n$. The conditional probability of $Y_{i}> \tau$, i.e., $\overset{̃}{Y_{i}}=1$ could be represented as a function of the estimated intercept and coefficients from linear model:

$$\hat{\Pr}\left( \overset{̃}{Y_{i}}=1 | E_{i},Z_{1i},\cdots,Z_{pi} \right)= \Phi\left( \frac{\hat{\alpha_{0}}-\tau+\hat{\alpha_{1}}E_{i}+\sum_{k=1}^{p} \hat{\alpha_{k+1}}Z_{ki}}{\hat{\lambda}} \right), (A3.1)$$

$\Phi(\cdot)$ is the cumulative density function for standard normal (or logistic) distribution. Using marginal standardization, RR could be estimated as below:

$$\hat{\mathrm{RR}}=\frac{\sum_{i=1}^{N} \hat{\Pr}(\overset{̃}{Y}=1|E=1,Z_{1}=z_{1i},\cdots,Z_{p}=z_{pi} )w_{i}}{\sum_{i=1}^{N} \hat{\Pr}(\overset{̃}{Y}=1|E=0,Z_{1}=z_{1i},\cdots,Z_{p}=z_{pi} )w_{i}}. (A3.2)$$

where the target population is the study population when $w_{i}=\frac{1}{n}$. Given that the scale parameter estimated from the linear model is on the log-scale, we replace $\hat{\lambda}$ with $exp(\hat{s})$ where $\hat{s}$ is the log-scale estimate.

Using delta method and equation (A3.2), $\hat{\mathrm{RR}}=g(\hat{\alpha},\hat{s})$, is also asymptotically normally distributed with mean corresponding to $g\left( \alpha,s \right)$, and the covariance matrix corresponding to ${g'\left( \alpha,s \right)}^{T}\boldsymbol{\Sigma}g^{'}\left( \alpha,s \right)$, where ${\hat{\alpha}=\left( \hat{\alpha_{0}},\ldots,\hat{\alpha_{p+1}} \right)}^{T}$, ${\alpha=\left( \alpha_{0},\ldots,\alpha_{p+1} \right)}^{T}$, $\boldsymbol{\Sigma}$ is the covariance matrix of $\left( \hat{\alpha},\hat{s} \right)^{T}$ and $g^{'}(\cdot)$ represents the first-order partial derivative of $g(\cdot)$ ^24^. The $\hat{\mathrm{RR}}$can be explicitly expressed by the MLEs of $\alpha_{j}$ and $s$ (i.e., $\hat{\alpha}_{j},\hat{s}$).

Let

$$\hat{lp}_{1i}=\hat{\alpha_{0}}-\tau+\hat{\alpha_{1}}+\sum_{k=1}^{p} \hat{\alpha_{k+1}}Z_{ki}$$

$$\hat{lp}_{0i}=\hat{\alpha_{0}}-\tau+\sum_{k=1}^{p} \hat{\alpha_{k+1}}Z_{ki}$$

$$\hat{\lambda}=exp\left( \hat{s} \right), and w_{i}=\frac{1}{n}$$

Then

$$\frac{\partial\hat{\mathrm{RR}}}{\partial\hat{\alpha_{0}}}=\frac{\left( \underset{i=1}{\overset{n}{\sum}}\frac{1}{\hat{\lambda}}\phi(\frac{\hat{lp}_{1i}}{\hat{\lambda}}) \right)\left( \underset{i=1}{\overset{n}{\sum}}\Phi(\frac{\hat{lp}_{0i}}{\hat{\lambda}}) \right)-\left( \underset{i=1}{\overset{n}{\sum}}\frac{1}{\hat{\lambda}}\phi(\frac{\hat{lp}_{0i}}{\hat{\lambda}}) \right)\left( \underset{i=1}{\overset{n}{\sum}}\Phi(\frac{\hat{lp}_{1i}}{\hat{\lambda}}) \right)}{\left( \underset{i=1}{\overset{n}{\sum}}\Phi(\frac{\hat{lp}_{0i}}{\hat{\lambda}}) \right)^{2}}$$

where $\phi(\cdot)$ represents standard normal (or logistic) probability density function,

$$\frac{\partial\hat{\mathrm{RR}}}{\partial\hat{\alpha_{1}}}=\frac{\left( \underset{i=1}{\overset{n}{\sum}}\frac{1}{\hat{\lambda}}\phi(\frac{\hat{lp}_{1i}}{\hat{\lambda}}) \right)}{\left( \underset{i=1}{\overset{n}{\sum}}\Phi(\frac{\hat{lp}_{0i}}{\hat{\lambda}}) \right)}$$

$$\frac{\partial\hat{\mathrm{RR}}}{\partial\hat{\alpha_{k+1}}}=\frac{\left( \underset{i=1}{\overset{n}{\sum}}\frac{z_{ki}}{\hat{\lambda}}\phi(\frac{\hat{lp}_{1i}}{\hat{\lambda}}) \right)\left( \underset{i=1}{\overset{n}{\sum}}\Phi(\frac{\hat{lp}_{0i}}{\hat{\lambda}}) \right)-\left( \underset{i=1}{\overset{n}{\sum}}\frac{z_{ki}}{\hat{\lambda}}\phi(\frac{\hat{lp}_{0i}}{\hat{\lambda}}) \right)\left( \underset{i=1}{\overset{n}{\sum}}\Phi(\frac{\hat{lp}_{1i}}{\hat{\lambda}}) \right)}{\left( \underset{i=1}{\overset{n}{\sum}}\Phi(\frac{\hat{lp}_{0i}}{\hat{\lambda}}) \right)^{2}}$$

where $k=1,\ldots,p$

$$\frac{\partial\hat{\mathrm{RR}}}{\partial\hat{s}}=\frac{\left( \underset{i=1}{\overset{n}{\sum}}(-\frac{\hat{lp}_{1i}}{\hat{\lambda}})\phi(\frac{\hat{lp}_{1i}}{\hat{\lambda}}) \right)\left( \underset{i=1}{\overset{n}{\sum}}\Phi(\frac{\hat{lp}_{0i}}{\hat{\lambda}}) \right)-\left( \underset{i=1}{\overset{n}{\sum}}(-\frac{\hat{lp}_{0i}}{\hat{\lambda}})\phi(\frac{\hat{lp}_{0i}}{\hat{\lambda}}) \right)\left( \underset{i=1}{\overset{n}{\sum}}\Phi(\frac{\hat{lp}_{1i}}{\hat{\lambda}}) \right)}{\left( \underset{i=1}{\overset{n}{\sum}}\Phi(\frac{\hat{lp}_{0i}}{\hat{\lambda}}) \right)^{2}}.$$

The derivation for RR estimates obtained from probit and logit models are similar to that of linear models with $\hat{\lambda}$ replaced by 1, $\tau$ being 0 and only first partial derivatives for $\hat{\beta}$s.

In the case when the dichotomized outcome is defined as falling below a threshold, i.e., $\tilde{Y}_{i}=I\left( Y_{i}<\tau\right)$, the conditional probability becomes:

$$\hat{\Pr}\left( \overset{̃}{Y_{i}}=1 | E_{i},Z_{1i},\cdots,Z_{pi} \right)= \Phi\left( -\frac{\hat{\alpha_{0}}-\tau+\hat{\alpha_{1}}E_{i}+\sum_{k=1}^{p} \hat{\alpha_{k+1}}Z_{ki}}{\hat{\lambda}} \right), (A3.3)$$

and the corresponding partial derivatives are as follows:

$$\frac{\partial\hat{\mathrm{RR}}}{\partial\hat{\alpha_{0}}}=\frac{\left( -\sum_{i=1}^{n} \frac{1}{\hat{\lambda}}\phi(-\frac{\hat{lp}_{1i}}{\hat{\lambda}}) \right)\left( \sum_{i=1}^{n} \Phi(\frac{\hat{lp}_{0i}}{\hat{\lambda}}) \right)-\left( -\sum_{i=1}^{n} \frac{1}{\hat{\lambda}}\phi(-\frac{\hat{lp}_{0i}}{\hat{\lambda}}) \right)\left( \sum_{i=1}^{n} \Phi(\frac{\hat{lp}_{1i}}{\hat{\lambda}}) \right)}{\left( \sum_{i=1}^{n} \Phi(\frac{\hat{lp}_{0i}}{\hat{\lambda}}) \right)^{2}}$$

where $\phi(\cdot)$ represents standard normal (or logistic) probability density function,

$$\frac{\partial\hat{\mathrm{RR}}}{\partial\hat{\alpha_{1}}}=\frac{\left( -\sum_{i=1}^{n} \frac{1}{\hat{\lambda}}\phi(-\frac{\hat{lp}_{1i}}{\hat{\lambda}}) \right)}{\left( \sum_{i=1}^{n} \Phi(\frac{\hat{lp}_{0i}}{\hat{\lambda}}) \right)}$$

$$\frac{\partial\hat{\mathrm{RR}}}{\partial\hat{\alpha_{k+1}}}=\frac{\left( -\sum_{i=1}^{n} \frac{z_{ki}}{\hat{\lambda}}\phi(-\frac{\hat{lp}_{1i}}{\hat{\lambda}}) \right)\left( \sum_{i=1}^{n} \Phi(\frac{\hat{lp}_{0i}}{\hat{\lambda}}) \right)-\left( -\sum_{i=1}^{n} \frac{z_{ki}}{\hat{\lambda}}\phi(-\frac{\hat{lp}_{0i}}{\hat{\lambda}}) \right)\left( \sum_{i=1}^{n} \Phi(\frac{\hat{lp}_{1i}}{\hat{\lambda}}) \right)}{\left( \sum_{i=1}^{n} \Phi(\frac{\hat{lp}_{0i}}{\hat{\lambda}}) \right)^{2}}$$

where $k=1,\ldots,p$

$$\frac{\partial\hat{\mathrm{RR}}}{\partial\hat{s}}=\frac{\left( \sum_{i=1}^{n} (\frac{\hat{lp}_{1i}}{\hat{\lambda}})\phi(-\frac{\hat{lp}_{1i}}{\hat{\lambda}}) \right)\left( \sum_{i=1}^{n} \Phi(\frac{\hat{lp}_{0i}}{\hat{\lambda}}) \right)-\left( \sum_{i=1}^{n} (\frac{\hat{lp}_{0i}}{\hat{\lambda}})\phi(-\frac{\hat{lp}_{0i}}{\hat{\lambda}}) \right)\left( \sum_{i=1}^{n} \Phi(\frac{\hat{lp}_{1i}}{\hat{\lambda}}) \right)}{\left( \sum_{i=1}^{n} \Phi(\frac{\hat{lp}_{0i}}{\hat{\lambda}}) \right)^{2}}$$

Similarly, the derivation for RR estimates obtained from probit and logit models are similar to that of linear models with $\hat{\lambda}$ replaced by 1, $\tau$ being 0 and only first partial derivatives for $\hat{\beta}$s required.

**SECTION 4: ADDITIONAL SIMULATION RESULTS**

**Simulation results when** $\boldsymbol{\lambda=0.5}$ **(i.e., standard deviation of the normal distribution and scale parameter of the logistic distribution are equal to 0.5)**

**Figure S1: Simulations results for bias, coverage probability and standard error for both** $\hat{\boldsymbol{\beta}_{\mathbf{1}}}$ **and** $\hat{\mathbf{RR}}$

Panel A and B plot the simulation results for $\hat{\beta_{1}}$, and panel C and D plot the simulation results for $\hat{\mathrm{RR}}$, when errors are normally (or logistically) distributed with mean (or location) 0 and standard deviation (or scale) 0.5 represented by Normal(0,0.5) (or Logistic(0,0.5)), and $\alpha_{1}=0$, $-0.15$, and -0.3. Horizontal dashed lines are 0, 0.95 and 1 for Bias, Coverage probability and Ratio: Mean/Empirical standard error (SE) respectively, which correspond to no bias, 95% coverage probability and mean and empirical SEs are the same. Normal and logistic linear models mean linear model with the error terms assumed to have normal and logistic distribution respectively.


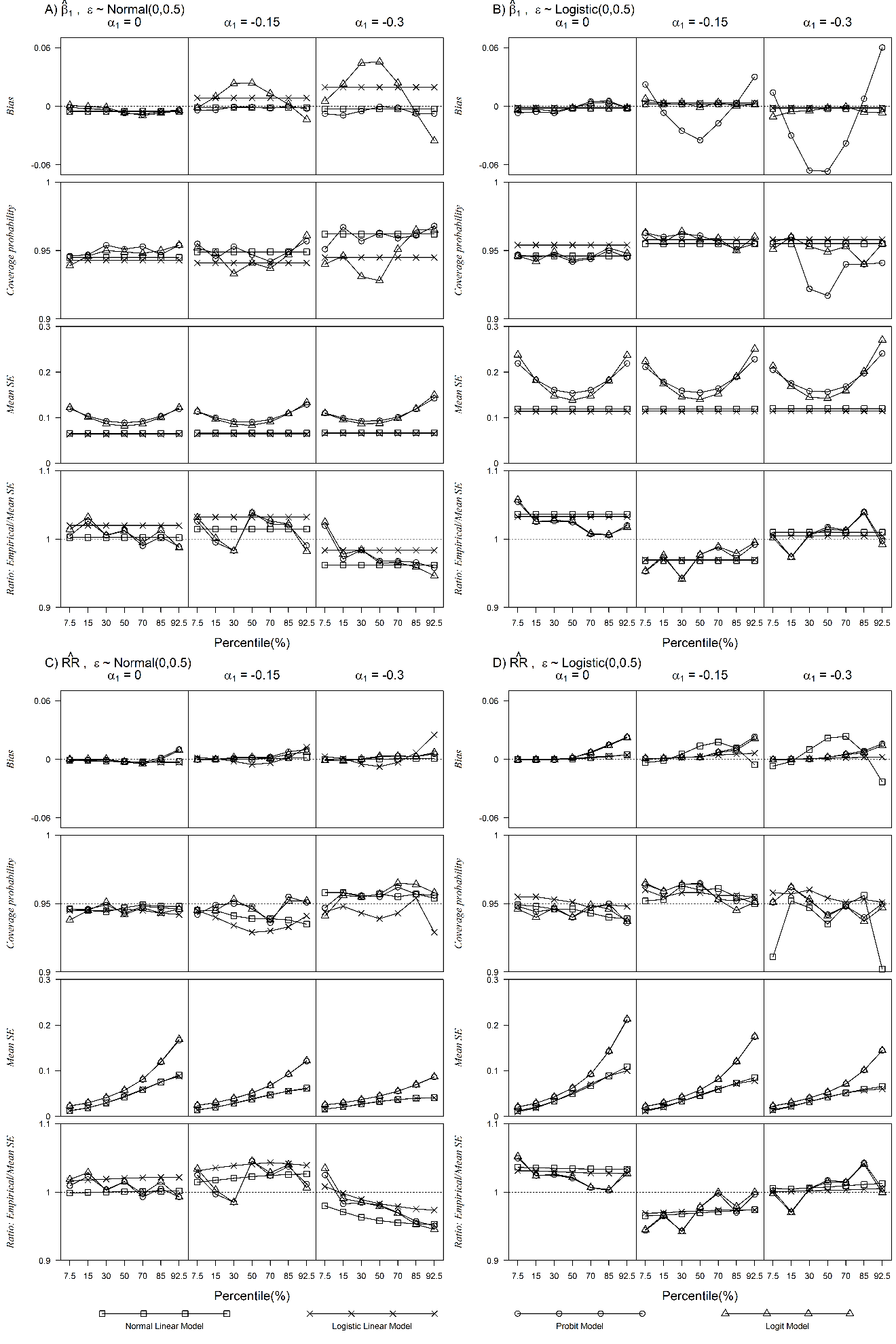


Figure S1

**Figure S2: Simulation results for type 1 error and power when** $\boldsymbol{\alpha}_{\mathbf{1}}\boldsymbol{=-0.15}$ **and** $\boldsymbol{\alpha}_{\mathbf{1}}\boldsymbol{=-0.3}$

Horizontal dashed lines are plotted at 0.05 and 1 for Type 1 error and Power respectively, which correspond to 0.05 type 1 error and 100% power. Normal and logistic linear models mean linear model with the error terms assumed to have normal and logistic distribution respectively, where Normal(0,0.5): Normal errors with mean 0 and standard deviation 0.5; and Logistic(0,0.5): Logistic errors with location 0 and scale 0.5


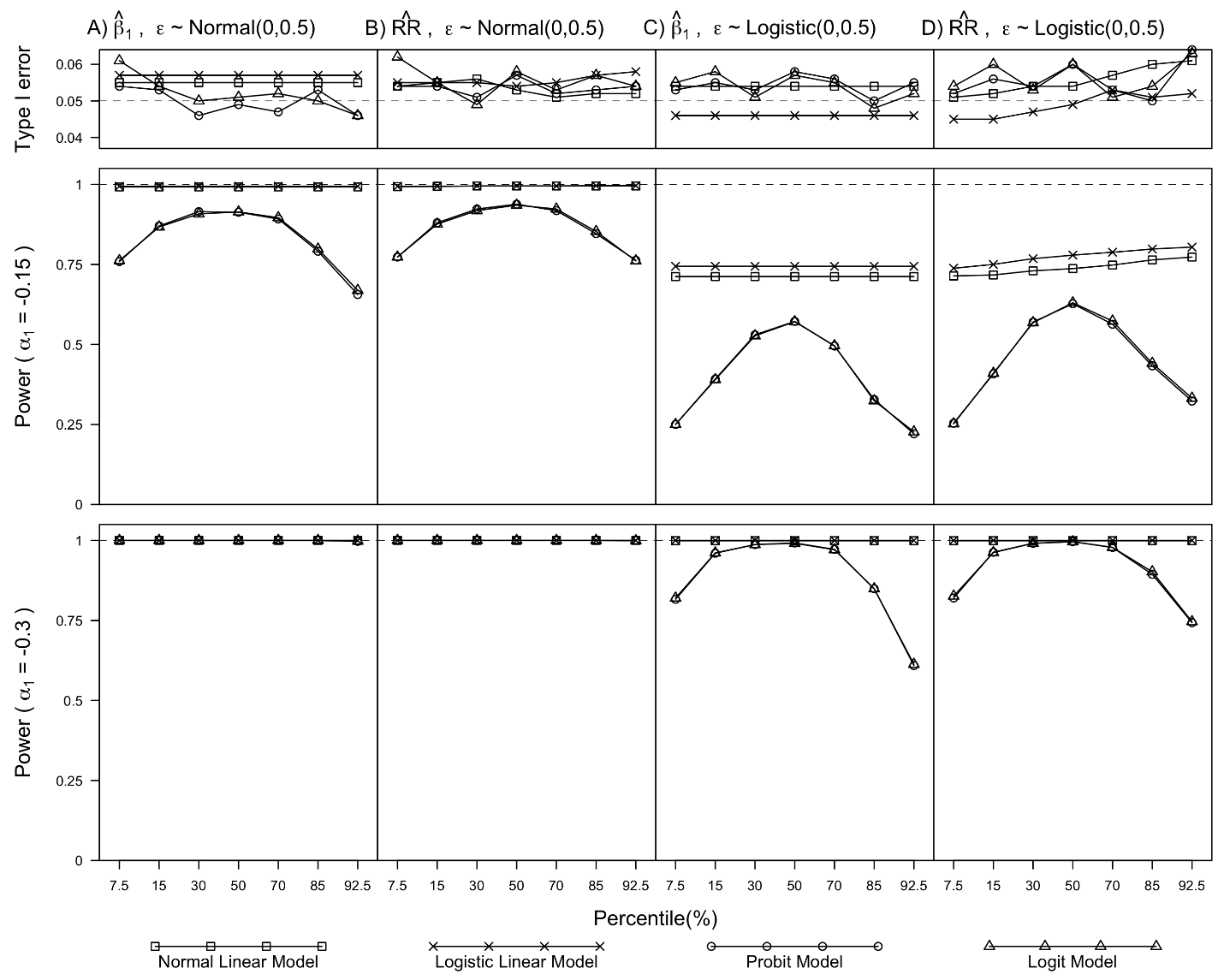


**Simulation results when** $\boldsymbol{\lambda=2}$ **(i.e., standard deviation of the normal distribution and scale parameter of the logistic distribution are equal to 2)**

**Figure S3: Simulations results for bias, coverage probability and standard error for both** $\hat{\boldsymbol{\beta}_{\mathbf{1}}}$ **and** $\hat{\mathbf{RR}}$

Panel A and B plot the simulation results for $\hat{\beta_{1}}$, and panel C and D plot the simulation results for $\hat{\mathrm{RR}}$, when errors are normally (or logistically) distributed with mean (or location) 0 and standard deviation (or scale) 2 represented by Normal(0,2) (or Logistic(0,2)), and $\alpha_{1}=0$, $-0.15$, and -0.3. Horizontal dashed lines are 0, 0.95 and 1 for Bias, Coverage probability and Ratio: Mean/Empirical standard error (SE) respectively, which correspond to no bias, 95% coverage probability and mean and empirical SEs are the same. Normal and logistic linear models mean linear model with the error terms assumed to have normal and logistic distribution respectively.


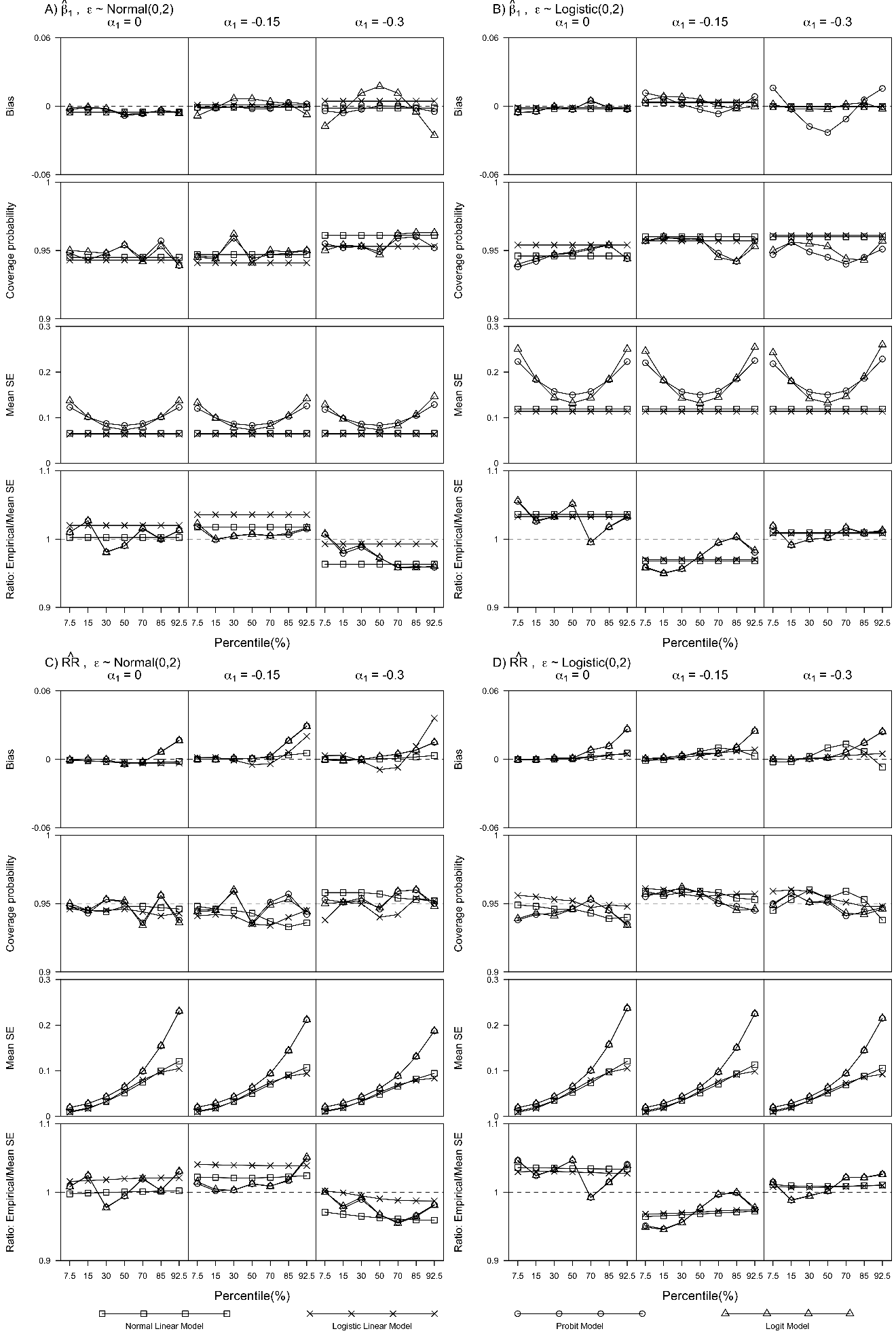


Figure S3

**Figure S4: Simulation results for type 1 error and power when** $\boldsymbol{\alpha}_{\mathbf{1}}\mathbf{=-0.15}$ **and** $\boldsymbol{\alpha}_{\mathbf{1}}\mathbf{=-0.3}$

Horizontal dashed lines are plotted at 0.05 and 1 for Type 1 error and Power respectively, which correspond to 0.05 type 1 error and 100% power. Normal and logistic linear models mean linear model with the error terms assumed to have normal and logistic distribution respectively, where Normal(0,2): Normal errors with mean 0 and standard deviation 2; and Logistic(0,2): Logistic errors with location 0 and scale 2.


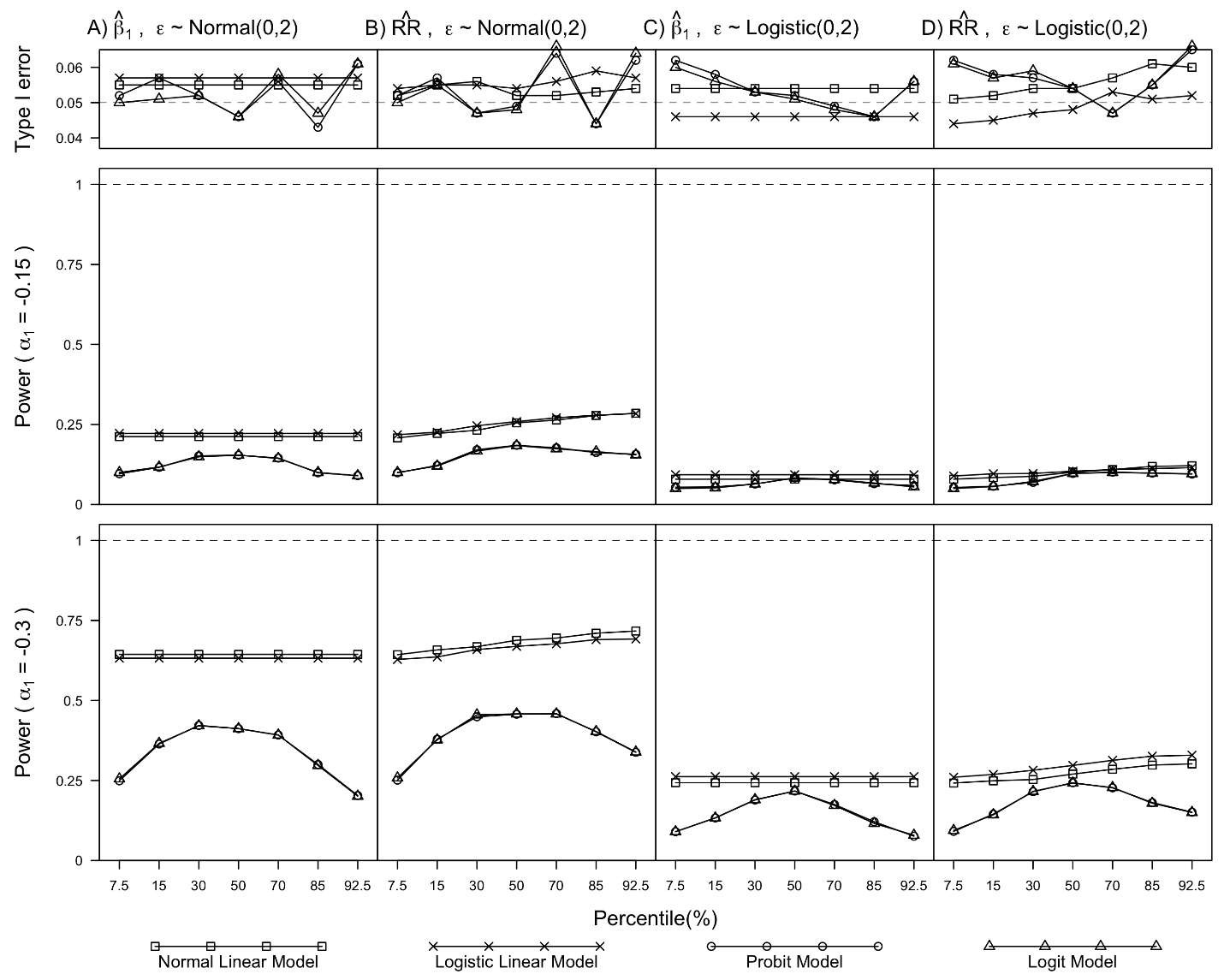


**Simulation results comparing extended marginal standardised method with marginal means method (*distdicho*)**

**Figure S5: Simulations results comparing extended marginal standardised method with marginal means method (*distdicho*) for** $\hat{\mathbf{RR}}$ **for data with normal error distribution where mean is 0 and standard deviation is 1**

For panel A, B and D, horizontal dashed lines are 0, 0.95 and 1 for Bias, Coverage probability and Ratio: Mean/Empirical standard error (SE) respectively, which correspond to no bias, 95% coverage probability and mean and empirical SEs are the same. For panel E, horizontal dashed lines are at 0.05 and 1 which correspond to 0.05 type 1 error when $\alpha_{1}=0$and 100% power when $\alpha_{1}\neq0$. Normal linear model means linear model with the error terms assumed to have normal distribution.

**
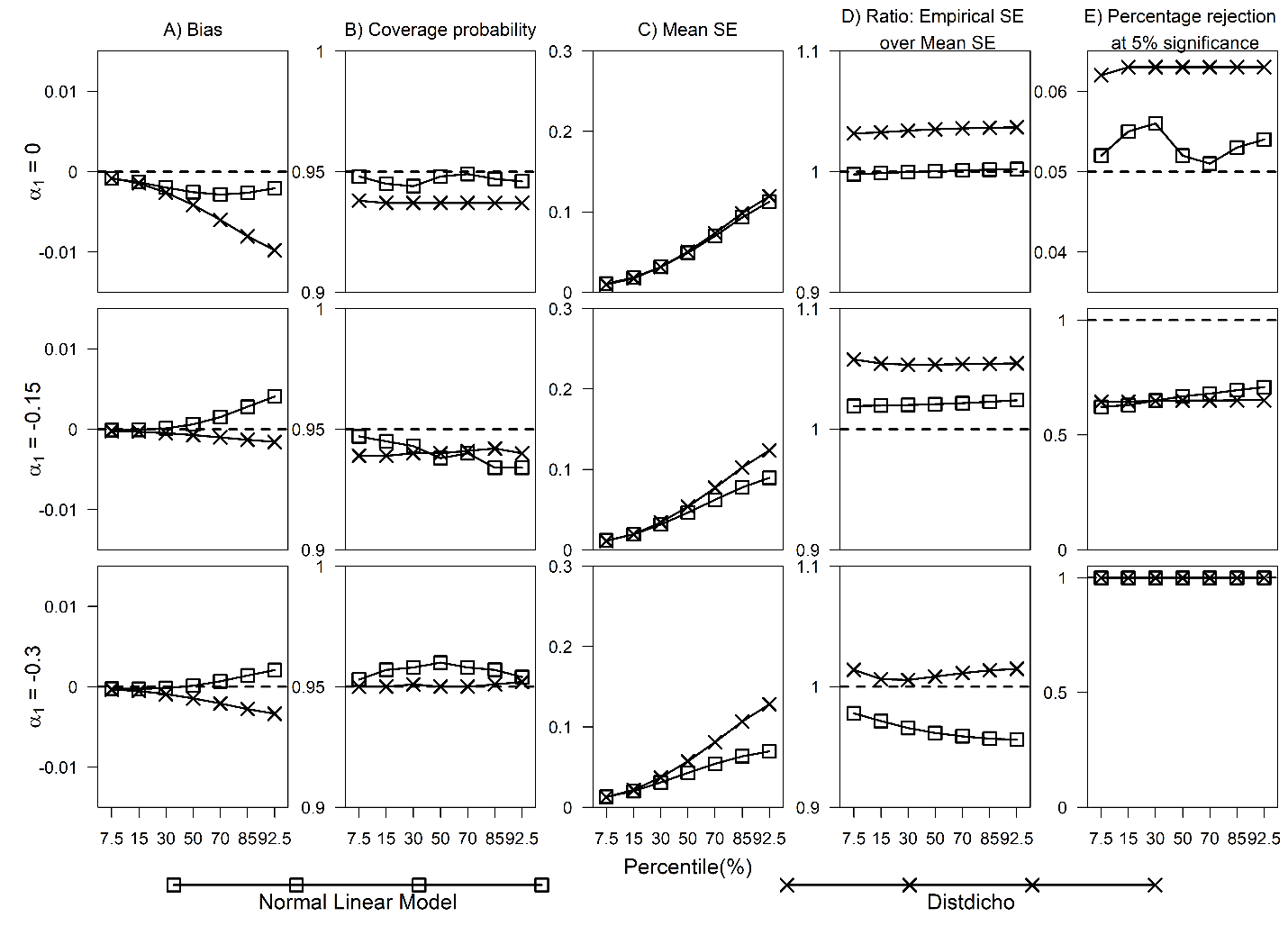
**

**Figure S6: Simulations results comparing extended marginal standardised method with marginal means method (*distdicho*) for** $\hat{\mathbf{RR}}$ **for data with normal error distribution where mean is 0 and standard deviation is 0.5**

For panel A, B and D, horizontal dashed lines are 0, 0.95 and 1 for Bias, Coverage probability and Ratio: Mean/Empirical standard error (SE) respectively, which correspond to no bias, 95% coverage probability and mean and empirical SEs are the same. For panel E, horizontal dashed lines are at 0.05 and 1, which correspond to 0.05 type 1 error when $\alpha_{1}=0$ and 100% power when $\alpha_{1}\neq0$. Normal linear model means linear model with the error terms assumed to have normal distribution.

**
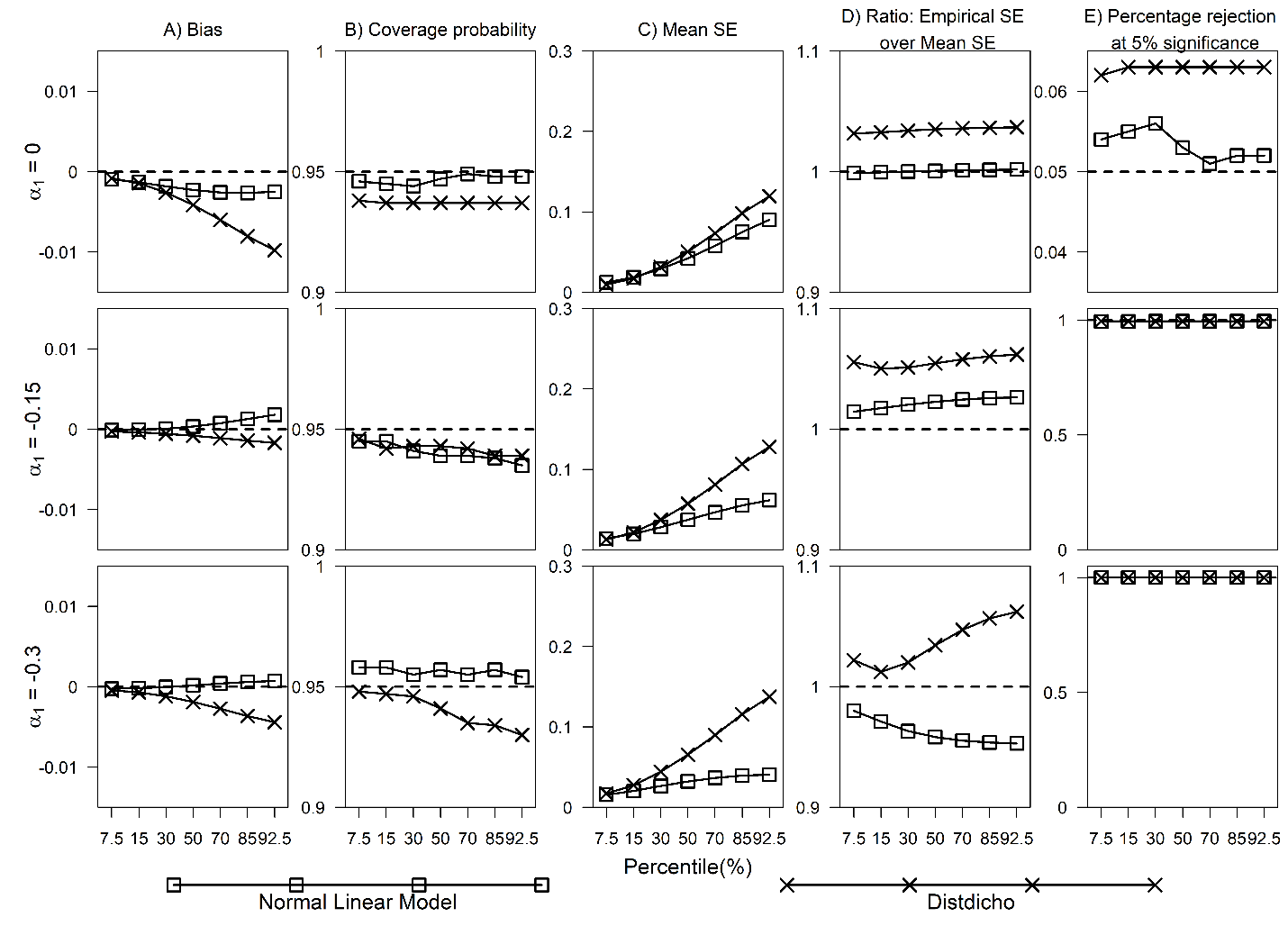
**

**Figure S7: Simulations results comparing extended marginal standardised method with marginal means method (*distdicho*) for** $\hat{\mathbf{RR}}$ **for data with normal error distribution where mean is 0 and standard deviation is 2**

For panel A, B and D, horizontal dashed lines are 0, 0.95 and 1 for Bias, Coverage probability and Ratio: Mean/Empirical standard error (SE) respectively, which correspond to no bias, 95% coverage probability and mean and empirical SEs are the same. For panel E, horizontal dashed lines are at 0.05 and 1, which correspond to 0.05 type 1 error when $\alpha_{1}=0$ and 100% power when $\alpha_{1}\neq0$. Normal linear model means linear model with the error terms assumed to have normal distribution.

**
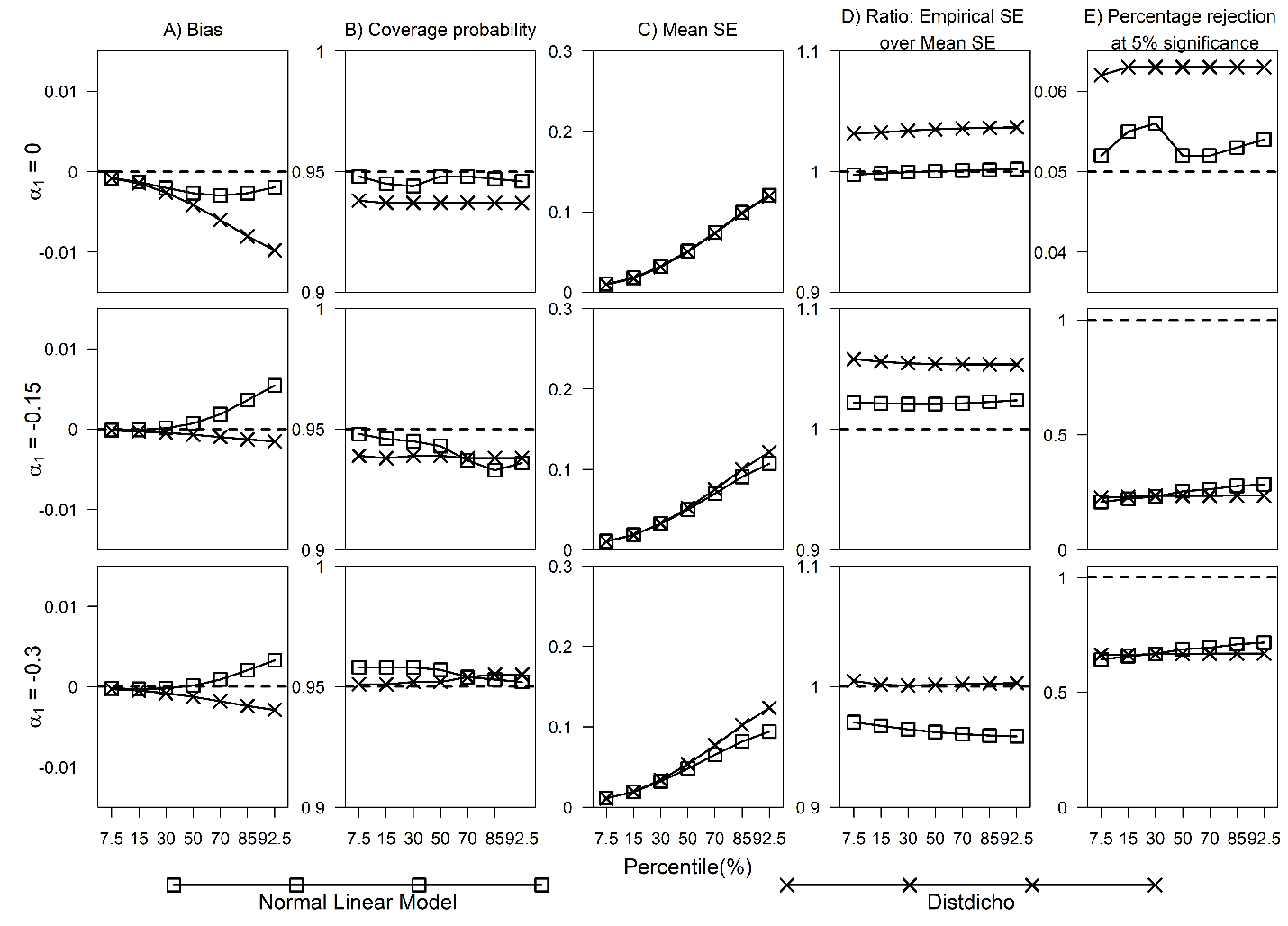
**
